# Variable DPP4 expression in multiciliated cells of the human nasal epithelium as a determinant for MERS-CoV tropism

**DOI:** 10.1073/pnas.2410630122

**Published:** 2025-03-06

**Authors:** Tim I. Breugem, Samra Riesebosch, Jingshu Zhang, Anna Z. Mykytyn, Lisette Krabbendam, Nathalie Groen, Sivana Baptista Varela, Debby Schipper, Petra B. van den Doel, Romy van Acker, Ralph Stadhouders, Mart M. Lamers, Bart L. Haagmans

**Affiliations:** ^a^Viroscience Department, Erasmus University Medical Center, Rotterdam 3015 GD, The Netherlands; ^b^Pulmonary Medicine Department, Erasmus University Medical Center, Rotterdam 3015 GD, The Netherlands; ^c^Single Cell Discoveries, Utrecht 3584 BW, The Netherlands; ^d^Programme in Emerging Infectious Diseases, Duke-National University of Singapore Medical School, Singapore 169857, Singapore

**Keywords:** MERS-CoV, coronavirus, nose, organoids, DPP4

## Abstract

Transmissibility of respiratory viruses is a complex viral trait that is intricately linked to tropism. Several highly transmissible viruses, including severe acute respiratory syndrome coronavirus 2 and Influenza viruses, specifically target multiciliated cells in the upper respiratory tract to facilitate efficient human-to-human transmission. In contrast, the zoonotic Middle East respiratory syndrome coronavirus (MERS-CoV) generally transmits poorly between humans, which is largely attributed to the absence of its receptor dipeptidyl peptidase 4 (DPP4) in the upper respiratory tract. At the same time, MERS-CoV epidemiology is characterized by occasional superspreading events, suggesting that some individuals can disseminate this virus effectively. Here, we utilized well-differentiated human pulmonary and nasal airway organoid-derived cultures to further delineate the respiratory tropism of MERS-CoV. We find that MERS-CoV replicated to high titers in both pulmonary and nasal airway cultures. Using single-cell messenger-RNA sequencing, immunofluorescence, and immunohistochemistry, we show that MERS-CoV preferentially targeted multiciliated cells, leading to loss of ciliary coverage. MERS-CoV cellular tropism was dependent on the differentiation of the organoid-derived cultures, and replication efficiency varied considerably between donors. Similarly, variable and focal expression of DPP4 was revealed in human nose tissues. This study indicates that the upper respiratory tract tropism of MERS-CoV may vary between individuals due to differences in DPP4 expression, providing an explanation for the unpredictable transmission pattern of MERS-CoV.

## Significance

Middle East respiratory syndrome coronavirus (MERS-CoV) is a highly pathogenic coronavirus that continues to cause periodic outbreaks in humans with a case-fatality rate of approximately 35%. MERS-CoV generally transmits poorly, but superspreading events are well documented. Efficient human-to-human transmission of respiratory viruses generally correlates with a tropism for the upper respiratory tract, but this tropism for MERS-CoV remains poorly understood. Characterizing the MERS-CoV tropism in the human upper respiratory tract is of critical importance to understand its epidemiology and pandemic potential of future MERS-CoV variants and other dipeptidyl peptidase 4 (DPP4)-utilizing coronaviruses present in animal reservoirs.

Middle East respiratory syndrome coronavirus (MERS-CoV) is a highly pathogenic betacoronavirus, responsible for recurrent outbreaks in the Arabian Peninsula with a case-fatality rate of approximately 35% (1). MERS-CoV was first identified in a patient with severe pneumonia in the Kingdom of Saudi Arabia in 2012 (2). To date, over 2,500 laboratory-confirmed MERS-CoV cases have been reported (1), which are associated with male sex [69,4%] and a median age of 53 y (3). Older age and underlying medical conditions are the main predictors of severe disease and mortality during MERS-CoV infection (4–6). Although the virus circulates in dromedary camels in various geographical regions (7–9), zoonotic spillover events to humans remain relatively rare. Most laboratory-confirmed MERS-CoV cases are concentrated in high-risk groups with close camel contacts or healthcare workers (10, 11). Furthermore, sustained human-to-human transmission is inefficient for MERS-CoV and seems mostly limited to healthcare settings (12–14). The basic reproduction number R of MERS-CoV during outbreak scenarios in the Middle East generally does not exceed 1 and secondary attack rates are low, ranging from 0.43 to 4%, resulting in limited transmission generations (15–19). Only in rare situations, such as the large nosocomial outbreak of 2015 in the Republic of South Korea, does MERS-CoV achieve reproduction numbers [2.5 to 8.09] and secondary attack rates [3.7 to 15.8%] associated with sustained human-to-human transmission (20). Interestingly, these large-scale MERS-CoV nosocomial outbreaks have been associated with superspreading events (21–25). At the same time, a significant proportion of primary MERS-CoV cases [17.11 to 37.5%] are not associated with camel contacts (10, 26, 27), and seroprevalence studies in cohorts without reported infections indicate that mild or asymptomatic MERS-CoV infections do occur (28, 29). These observations suggest that some MERS-CoV-infected individuals can transmit the virus efficiently.

Efficient transmission of respiratory viruses, including the pandemic severe acute respiratory syndrome coronavirus 2 (SARS-CoV-2) and Influenza viruses, is often associated with a tropism for multiciliated cells of the upper respiratory tract (30–32). On the other hand, MERS-CoV has been shown previously to target mostly nonciliated cells and pneumocytes of the lower lungs resulting in severe disease (33–35). Coronavirus tropism is guided by the distribution and expression of essential viral entry factors in host tissues. The SARS-CoV-2 receptor angiotensin-converting enzyme 2 (ACE2) is efficiently expressed in the ciliated epithelium of the upper respiratory tract and nose in humans (36–38). ACE2 expression significantly decreases throughout the lower respiratory tract (37), correlating with SARS-CoV-2’s preferential upper respiratory tract tropism. On the other hand, there is a current consensus that in humans the MERS-CoV receptor, dipeptidyl peptidase 4 (DPP4), is mostly expressed in the secretory cells, alveolar pneumocytes and alveolar macrophages of the lung (39–41), whereas DPP4 is considered absent on the ciliated epithelium of the upper respiratory tract and nose (41, 42). Therefore, the upper respiratory tract tropism of MERS-CoV seems limited to highly susceptible animals that express DPP4 in this anatomical region, such as dromedary camels (42), alpacas (43), and llama’s (44, 45). In these animals, the DPP4 expression pattern is associated with efficient viral dissemination (44, 46–51). Further unraveling the tropism of MERS-CoV in the human respiratory tract is essential to understand its transmission dynamics and epidemiology in humans. Here, we characterize the MERS-CoV tropism for the human respiratory tract by utilizing well-differentiated human organoid-derived air–liquid interface (ALI) cultures from the pulmonary airway and nasal airway epithelium.

## Results

### Single-Cell mRNA Sequencing of MERS-CoV-Infected Pulmonary Airway Cultures.

It has previously been shown that MERS-CoV primarily targets nonciliated cells in the human pulmonary airways, however, the exact identity of these target cells was poorly defined. To further delineate the cellular tropism of MERS-CoV in this manuscript, we utilized human small and large pulmonary airway organoid-derived epithelial cultures (SAECs and LAECs) that were differentiated at the air–liquid interface on transwell inserts for at least 6 wk (*SI Appendix*, Fig. S1 *A* and *B*). These cultures were used previously by our group (52, 53). All experiments in this manuscript were performed with well-differentiated organoid-derived ALI cultures, unless specified otherwise. Immunohistochemistry (IHC) and immunofluorescent staining (IF) revealed that our cultures expressed DPP4 in goblet cells (MUC5AC^+^), club cells (SCGB1A1^+^), and in a subset of multiciliated cells (ACTUB^+^) (*SI Appendix*, Fig. S1 *C*–*F*). IF on formalin-fixed paraffin-embedded (FFPE) cross-sections of SAECs and LAECs were used to quantify the expression of DPP4 in these cell subsets (*SI Appendix*, Fig. S1 *G*–*P*). DPP4 was expressed in a minor subset of epithelial cells of the pulmonary airways (*SI Appendix*, Fig. S1*M*), with a roughly twofold difference between the SAECs and LAECs while cell type distributions were similar (*SI Appendix*, Fig. S1 *M*, *N*, and *P*). In the SAECs, ~10% of cells were MUC5AC^+^/DPP4^+^ or SCGB1A1^+^/DPP4^+^, and ~5% were FOXJ1^+^/DPP4^+^ (*SI Appendix*, Fig. S1*O*). In the LAECs, ~5% of cells were MUC5AC^+^/DPP4^+^ or SCGB1A1^+^/DPP4^+^, and ~2% were FOXJ1^+^/DPP4^+^ (*SI Appendix*, Fig. S1*Q*). These data show that DPP4 is expressed in a small population of cells in the pulmonary airway cultures and indicate that DPP4 is mostly expressed in the secretory subsets of the pulmonary epithelium.

**Fig. 1.**
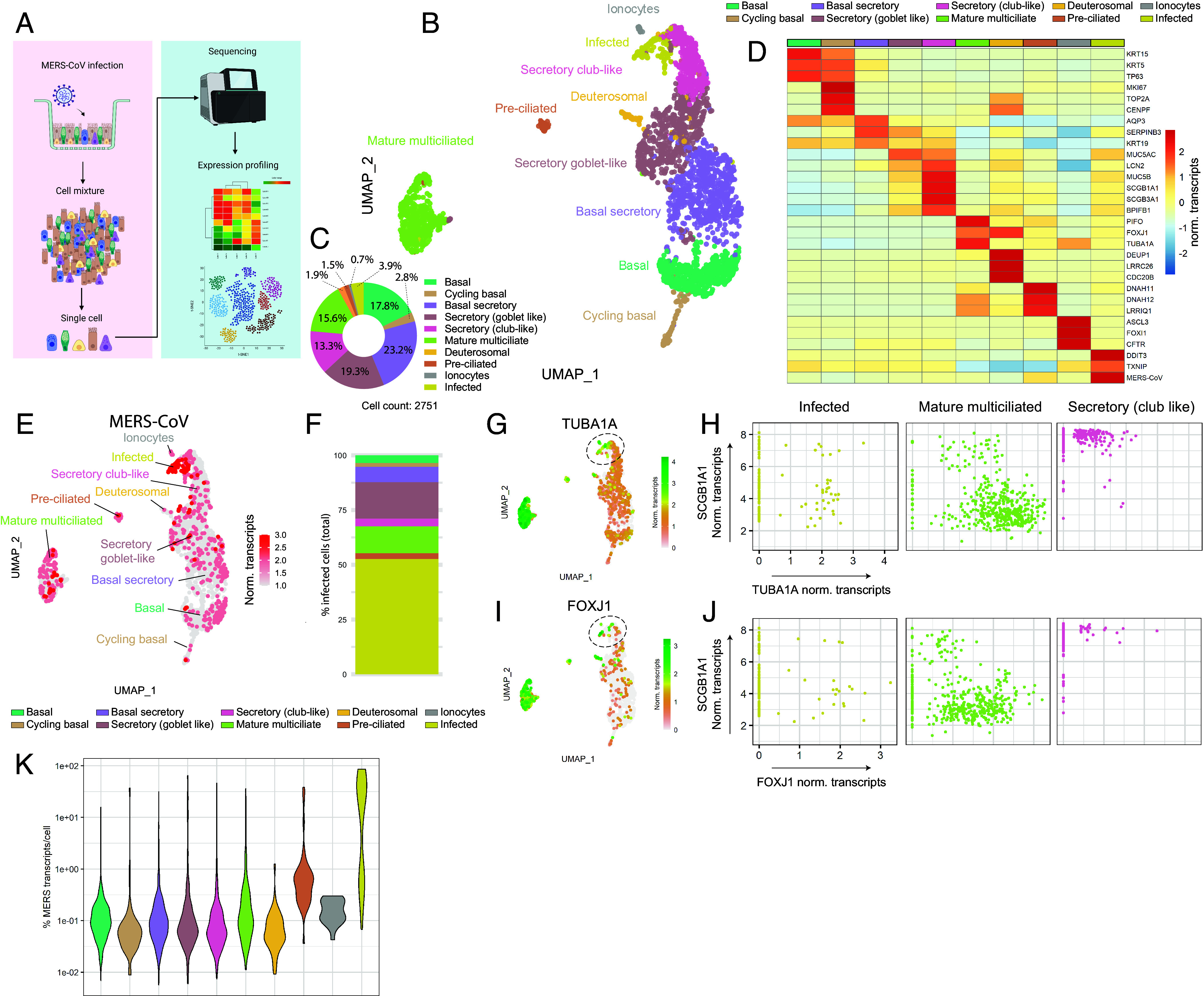
Single-cell mRNA sequencing of MERS-CoV-infected human pulmonary airway cultures. Small pulmonary airway organoid-derived cultures (SAECs) were infected with MERS-CoV at a moi of 1 and used at 1 dpi for 10× scRNA-seq to determine its cellular tropism. (*A*) Schematic of scRNA-seq experiment. Illustration was created with BioRender.com. (*B*) UMAP plot showing unsupervised clustering of MERS-CoV-infected SAECs. (*C*) Pie chart of relative proportion of all cell types identified in SAECs. (*D*) Heatmap of expression for top marker genes of each cluster. (*E*) UMAP plot showing normalized expression of MERS-CoV transcripts (>100 transcripts per cell) in all clusters. (*F*) Bar plot showing relative abundance of MERS-CoV-infected cells per cluster. (*G*) UMAP showing normalized expression of TUB1A1 transcripts in total clustered cells. (*H*) Scatter plots showing expression distribution of SCGB1A1 and TUB1A1 in the infected (*Left*), mature multiciliated (*Middle*), and secretory club-like (*Right*) clusters. (*I*) UMAP showing normalized expression of FOXJ1 transcripts in total clustered cells. (*J*) Scatter plots showing expression distribution of SCGB1A1 and FOXJ1 in the infected (*Left*), mature multiciliated (*Middle*), and secretory club-like (*Right*) clusters. (*K*) Violin plot showing the percentage of MERS-CoV transcripts from total transcripts per cell in each cluster. The gene counts were normalized for sequencing depth per cell, i.e., norm transcripts, and depicted on a log scale.

Next, we infected SAECs with MERS-CoV and performed 10× single-cell mRNA sequencing (scRNA-seq) after 24 h to investigate the cellular tropism of this virus (Fig. 1*A*). Unsupervised clustering distinguished 10 different cell clusters in our SAECs (Fig. 1 *B*–*D*). The bulk of the cells composed of basal cells [17.8%], basal-secretory cells [23.2%], secretory goblet-like cells [19.3%], secretory club-like cells [13.3%], and mature multiciliated cells [15.6%] (Fig. 1*C*). Minor cell subsets, such as the cycling basal cells [2.8%], deuterosomal cells [1.9%], ionocytes [0.7%], and preciliated cells [1.5%] were also identified (Fig. 1*C*). We detected transcripts of key coronavirus entry factors TMPRSS2, FURIN, and DPP4 in various subsets of cells, but transcript levels were generally low (*SI Appendix*, Fig. S2). Notably, DPP4 transcripts were only detected in the nonciliated cell subsets (*SI Appendix*, Fig. S2), in contrast to our protein expression data (*SI Appendix*, Fig. S1 *C*–*P*), indicating different molecular and regulatory dynamics between mRNA and protein levels of DPP4 in the airway cultures.

Next, we determined the transcriptional profiles of MERS-CoV-infected cells [>100 MERS-CoV transcripts, MERS-CoV^+^] (Fig. 1*E*). Roughly 20% of MERS-CoV^+^ cells were present in the secretory cell lineages (Fig. 1*F*). To our surprise, ~15% of MERS-CoV^+^ cells were concentrated in the mature ciliated clusters and ~55% of MERS-CoV^+^ cells were clustered together in one group, designated as the “infected” population (Fig. 1 *B*–*F*). The “infected” cluster was primarily defined by markedly high expression of MERS-CoV transcripts and ER stress markers (Fig. 1*D* and *SI Appendix*, Fig. S3*A*). Expression of interferon-related genes was minimal but correlated with the distribution of infected cells (*SI Appendix*, Fig. S3*B*). Interestingly, some cells of the infected population expressed key markers of the ciliated lineage, such as TUB1A1 and FOXJ1 (Fig. 1 *G*–*J* and *SI Appendix*, Fig. S4). However, this assessment was difficult to make, as up to 90% of transcripts in the “infected” cluster were actually MERS-CoV-related genes (Fig. 1*K*), limiting the detection of cell marker transcripts and convoluting cluster definition. Interestingly, DPP4 transcripts were not enriched in the “infected” population (*SI Appendix*, Fig. S2*B*). Taken together, these data showed that in addition to the known secretory cell tropism, cells of the multiciliated cell lineage might be a target for MERS-CoV infection in the human respiratory tract.

### MERS-CoV Mainly Targets Multiciliated Cells in Pulmonary Airway Cultures.

**Fig. 2.**
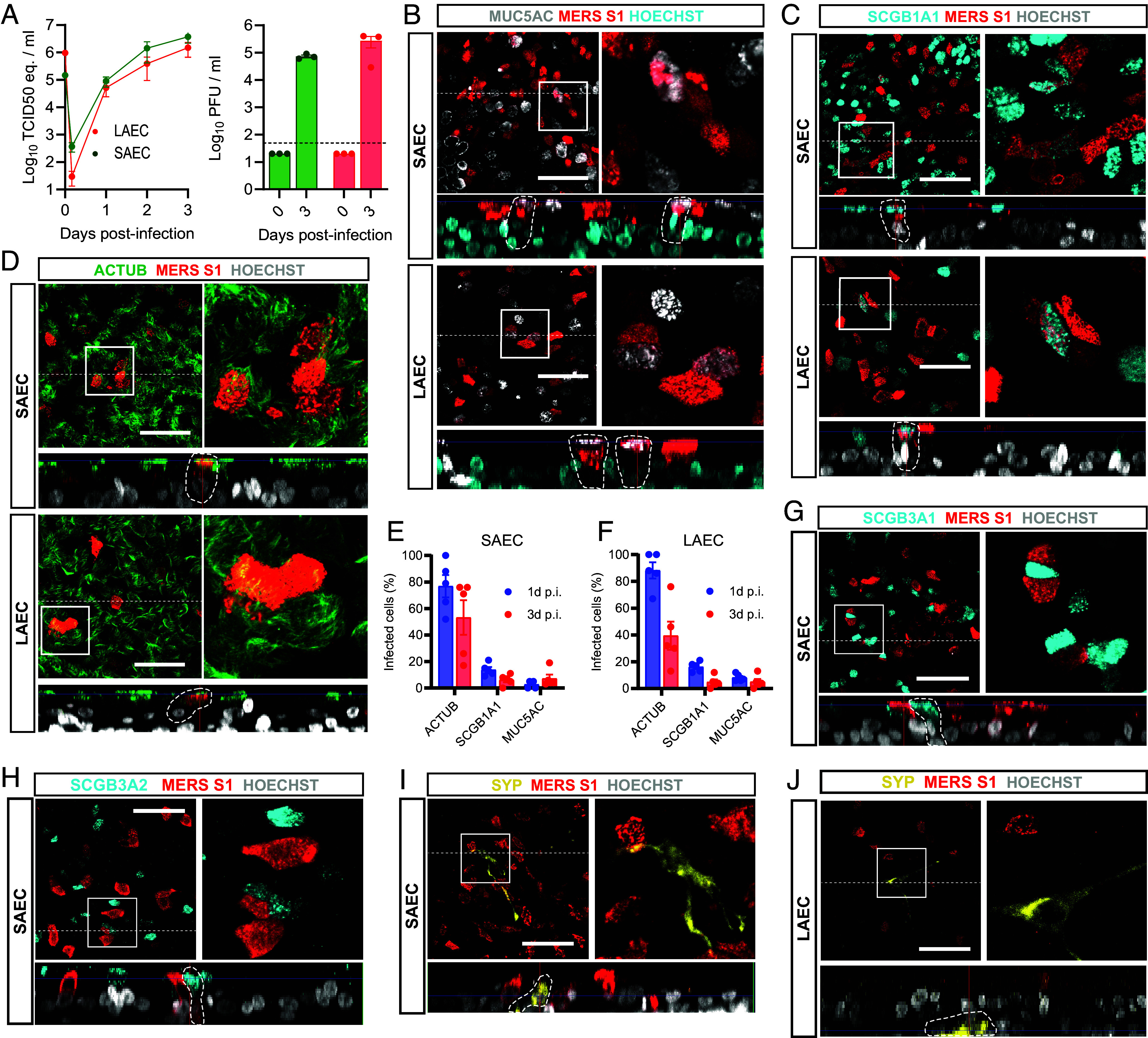
MERS-CoV mainly targets multiciliated cells in pulmonary airway cultures. Imaging experiments were conducted to further delineate the cellular tropism of MERS-CoV in SAECs and LAECs. (*A*) Human SAECs (green) and LAECs (red) were infected with MERS-CoV at a moi of 0.1, and virus was detected in the culture supernatant by RT-qPCR (*Left*) and plaque assay titration (*Right*). Data are shown as mean and SEM of three biological replicates from one representative experiment. The dotted line shows limit of detection. (*B*–*J*) SAECs and LAECs were infected with MERS-CoV at moi 1 and fixed after 1 dpi for IF, or at moi 0.1 and fixed after 3 dpi. (*B* and *C*) Infected cultures were stained at 1 dpi with MERS-S1 (red) and MUC5AC (white, B) and SCGB1A1 (cyan, C). (*D*) Infected SAECs and LAECs were stained at 1 dpi with MERS-S1 (red) and ACTUB (green). (*E* and *F*) Infected cells were quantified in the SAECs (*E*) and LAECs (*F*) at 1 dpi (blue) and 3 dpi (red). Data are shown as mean with SEM from five replicate images from one infected insert, each dot represents one replicate. (*G*–*I*) Infected SAECs were stained at 1 dpi with MERS-S1 (red) and SCGB3A1 (cyan, *H*), SCGB3A2 (cyan, *I*), and SYP (yellow, *J*). (*J*) Infected LAECs were stained at 1 dpi with MERS-S1 (red) and SYP (yellow). Experiments were repeated at least once. Representative images of each staining are shown. Nuclei (white or cyan) were stained with Hoechst. The dotted line indicates the position of the orthogonal view within the z-stack. (Scale bars represent 50 µm.) Inserts (squares) show digital zoom of the original image.

To further delineate the specific target cells for MERS-CoV in the respiratory tract we performed IF and IHC on MERS-CoV-infected SAECs and LAECs (Fig. 2). In the SAECs and LAECs, MERS-CoV replicated to equally high titers, reaching an infectious viral titer of ~10^5^ plaque-forming units (pfu) per mL at 3 days postinfection (dpi) after infection with a moi of 0.1 (Fig. 2*A*). We did not observe clear cytopathic effects in our MERS-CoV-infected SAECs and LAECs. The cells were infected with a moi of 1 or 0.1 and fixed at 1 or 3 dpi respectively and stained for viral antigen (MERS-CoV^+^) and various cellular markers. We demonstrated that at 1 dpi MUC5AC^+^ goblet cells and SCGB1A1^+^ club cells were infected with MERS-CoV (Fig. 2 *B* and *C*). However, a substantial population of MERS-CoV^+^ cells were ACTUB^+^ multiciliated cells (Fig. 2*D*). We quantified the MERS-CoV^+^ cells at 1 and 3 dpi by measuring costaining of viral antigen with markers of the multiciliated, goblet, and club cells (Fig. 2 *E* and *F*). At 1 dpi ~75% of infected cells were ACTUB^+^, whereas MUC5AC^+^ cells and SCGB1A1^+^ cells together composed of ~20% of MERS-CoV^+^ cells (Fig. 2 *E* and *F*). These data suggest that although DPP4 was more abundant on secretory cells in the SAECs and LAECs (*SI Appendix*, Fig. S1), the subset of DPP4^+^ multiciliated cells was preferentially targeted by MERS-CoV in the pulmonary airway epithelium during the early phase of infection. At 3 dpi the fraction of MERS-CoV^+^/ACTUB^+^ cells decreased to 40 to 50%, but the fractions of MERS-CoV^+^/MUC5AC^+^ and MERS-CoV^+^/SCGB1A1^+^ cells remained similar. Decreased ACTUB expression on infected cells might result from loss of ciliary coverage. MERS-CoV did not infect the minor nonciliated cell populations, such as SCGB3A1^+^ and SCGB3A2^+^ secretory cells or SYP^+^ neuroendocrine cells (Fig. 2 *G*–*J*). SCGB3A1 and SCGB3A2 were only expressed in SAECs (Fig. 2 *G* and *H*), not in LAECs. Overall, these data indicate that MERS-CoV infects mainly multiciliated cells in our pulmonary airway cultures.

We performed IF and IHC at 3 dpi to investigate the potential loss of ciliary coverage caused by MERS-CoV infection (*SI Appendix*, Fig. S5). We observed that MERS-CoV^+^ multiciliated cells had shorter cilia compared to noninfected neighboring cells (*SI Appendix*, Fig. S5 *A*–*D*). Quantification of cilia length at 3 dpi shows markedly shorter cilia for MERS-CoV-infected cells compared to uninfected cells in both LAECs and SAECs (*SI Appendix*, Fig. S5 *C* and *D*), which is indicative of MERS-CoV-induced ciliopathy. To investigate whether infection results in loss of ciliated cell identity, we stained for the key transcription factor FOXJ1 that is required for the formation of motile cilia, and indeed MERS-CoV^+^ cells were FOXJ1^dim^ (*SI Appendix*, Fig. S5 *E*–*H*). The dim FOXJ1 expression indicate that these cells still belong to the multiciliated cell lineage. These data suggest that MERS-CoV infection causes ciliopathy, possibly by the downregulation of FOXJ1 expression, which might contribute to pathogenesis by decreasing mucociliary clearance.

### MERS-CoV Cellular Tropism in Pulmonary Airway Cultures Depends on Differentiation but Not Donor Variation.

**Fig. 3.**
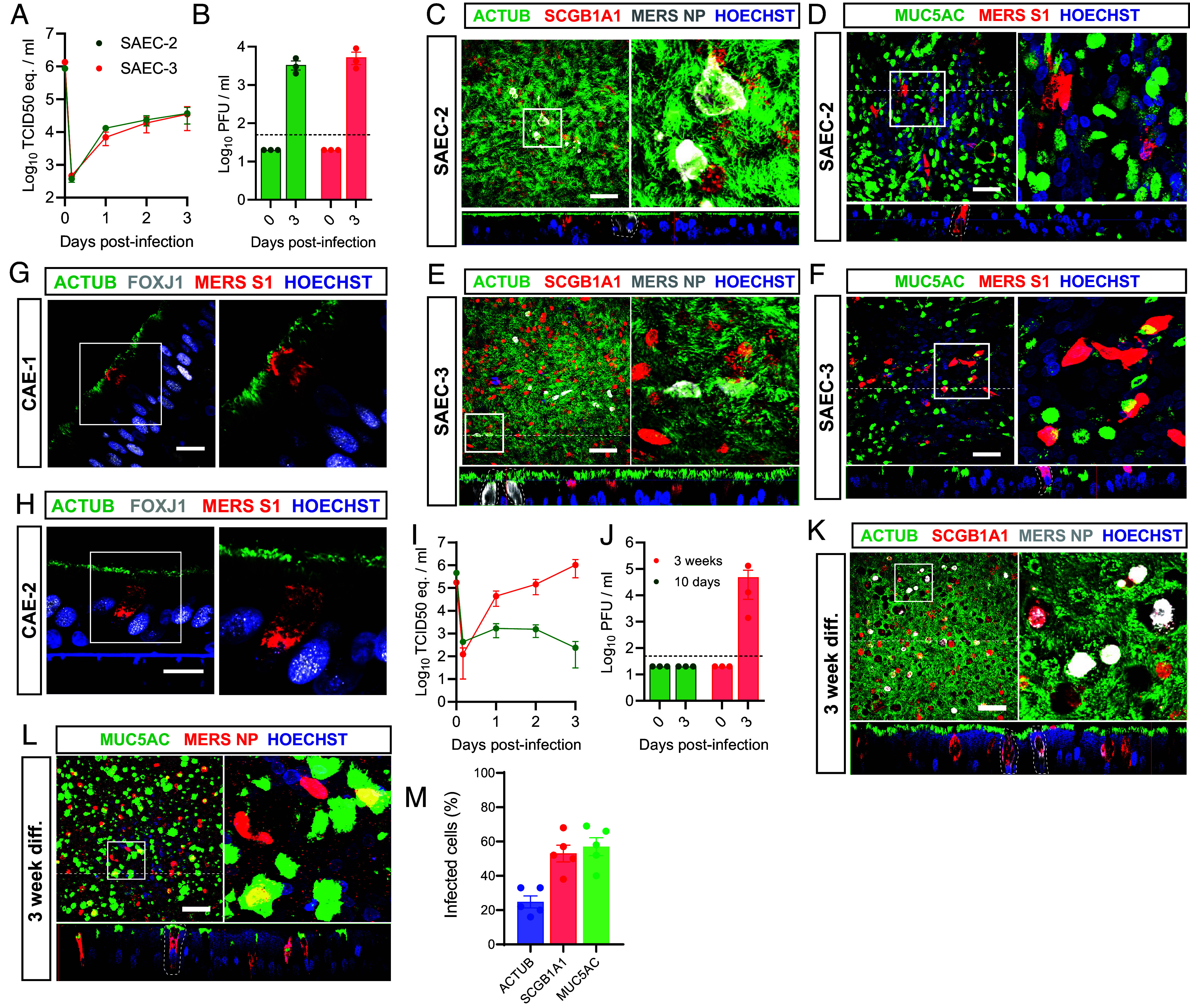
MERS-CoV cellular tropism in pulmonary airway cultures depends on differentiation but not donor variation. Imaging was used to determine whether the cellular tropism of MERS-CoV was affected by donor variation or differentiation times. (*A* and *B*) Human SAECs from two additional donors (SAEC-2 and SAEC-3) were infected with MERS-CoV at moi 0.1, and virus was detected in the culture supernatant by RT-qPCR (*A*) and plaque assay titration (*B*). Data are shown as mean and SEM from three biological replicates from one representative experiment. The dotted line shows limit of detection. (*C*–*F*) Cultures from SAEC-2 and SAEC-3 were infected with MERS-CoV at moi 0.1 and fixed at 3 dpi for IF. Imaging for ACTUB (green), SCGB1A1 (red), and MERS-NP (white) is shown for SAEC-2 (*C*) and SAEC-3 (*E*). Imaging for MUC5AC (green) and MERS-S1 (red) is shown for SAEC-2 (*D*) and SAEC-3 (*F*). (*G* and *H*) FFPE commercially available human airway epithelial cultures from two donors (CAE-1 and 2) were infected with MERS-CoV at moi 1 and fixed at 1 dpi and stained for ACTUB (green), FOXJ1 (white), and MERS-S1 (red). (*I* and *J*) Cultures from SAEC-1 were differentiated for 10 d or 3 wk and infected with MERS-CoV at moi 0.1, virus was detected in the culture supernatant by RT-qPCR (*I*) and plaque assay titration (*J*). Data are shown as mean and SEM of three biological replicates from one representative experiment. The dotted line shows limit of detection. (*K* and *L*) Infected SAEC-1 that were 3-wk differentiated were fixed at 3 dpi and imaged for (*K*) ACTUB (green), SCGB1A1 (red), and MERS-NP (white) or (*L*) MUC5AC (green) and MERS-NP (red). (*M*) Infected cells were quantified. Data are shown as mean with SEM from five replicate images from one infected insert, each dot represents one replicate. Experiments were repeated at least once. Representative images of each staining are shown. Nuclei (blue) were stained with Hoechst. The dotted line indicates the position of the orthogonal view within the z-stack. (Scale bars represent 50 µm.) Inserts (squares) show digital zoom of the original image.

To investigate whether the cellular tropism of MERS-CoV in the pulmonary airway is subject to donor differences, we included two additional organoid donors of SAECs (i.e., SAEC-2 and SAEC-3) (Fig. 3). Although MERS-CoV replicated to lower titers (~10^4^ pfu/mL), it targeted multiciliated cells in both donors (Fig. 3 *C*–*F*). In addition, we confirmed the multiciliated cell tropism of MERS-CoV in two commercially available small airway cultures (Fig. 3 *G* and *H*). Similar to our organoid-derived cultures, we observed MERS-CoV^+^/FOXJ1^dim^ cells in the commercial airway cultures from both donors (Fig. 3 *G* and *H*). These data indicate that the multiciliated cell tropism of MERS-CoV in the pulmonary airway is consistent across different donors.

In our scRNA-seq dataset, we previously demonstrated that DPP4 transcripts were restricted to few cells of the secretory lineages of our SAECs (*SI Appendix*, Fig. S2*B*). However, we could detect DPP4 at the protein level in both nonciliated and multiciliated cells (*SI Appendix*, Fig. S1). Given that multiciliated cells originate from the secretory lineages (54–58), DPP4 expression could be linked to the differentiation of the cultures, potentially affecting MERS-CoV tropism. To test this hypothesis, we differentiated SAECs for 10 d and 3 wk, respectively, and subsequently infected the cells with MERS-CoV at a moi of 0.1 (Fig. 3 *I*–*M*). Differentiation for 10 d was not sufficient for productive infection, while MERS-CoV effectively replicated in the 3-wk differentiated cells (Fig. 3 *I* and *J*). The tropism of MERS-CoV in the 3-wk differentiated cultures was more variable and shifted toward secretory cells (Fig. 2 *K* and *L*), compared to the consistent multiciliated cell tropism observed in at least 6-wk differentiated cultures shown previously (Fig. 2 *F* and *G*). In some experiments, ~50% of the infected cells were MUC5AC^+^ and SCGB1A1^+^, whereas only ~20% were ACTUB^+^ (Fig. 3*M*). IHC and IF on cultures from the same experiment showed lower DPP4 expression in the 3-wk differentiated cultures compared to 6-wk differentiated cultures (*SI Appendix*, Figs. S1 and S6). Less than 1% of cells coexpress DPP4 and FOXJ1 in these 3-wk differentiated cultures (*SI Appendix*, Fig. S6 *A*–*E*). Quantification of the cell subsets in these cultures revealed no clear differences (*SI Appendix*, Figs. S1*N* and S6*F*). Taken together, these data indicate that DPP4 expression in multiciliated cells might be affected by the differentiation status of pulmonary airway cultures.

### MERS-CoV Replicates Efficiently in Multiciliated Cells of Nasal Airway Cultures.

**Fig. 4.**
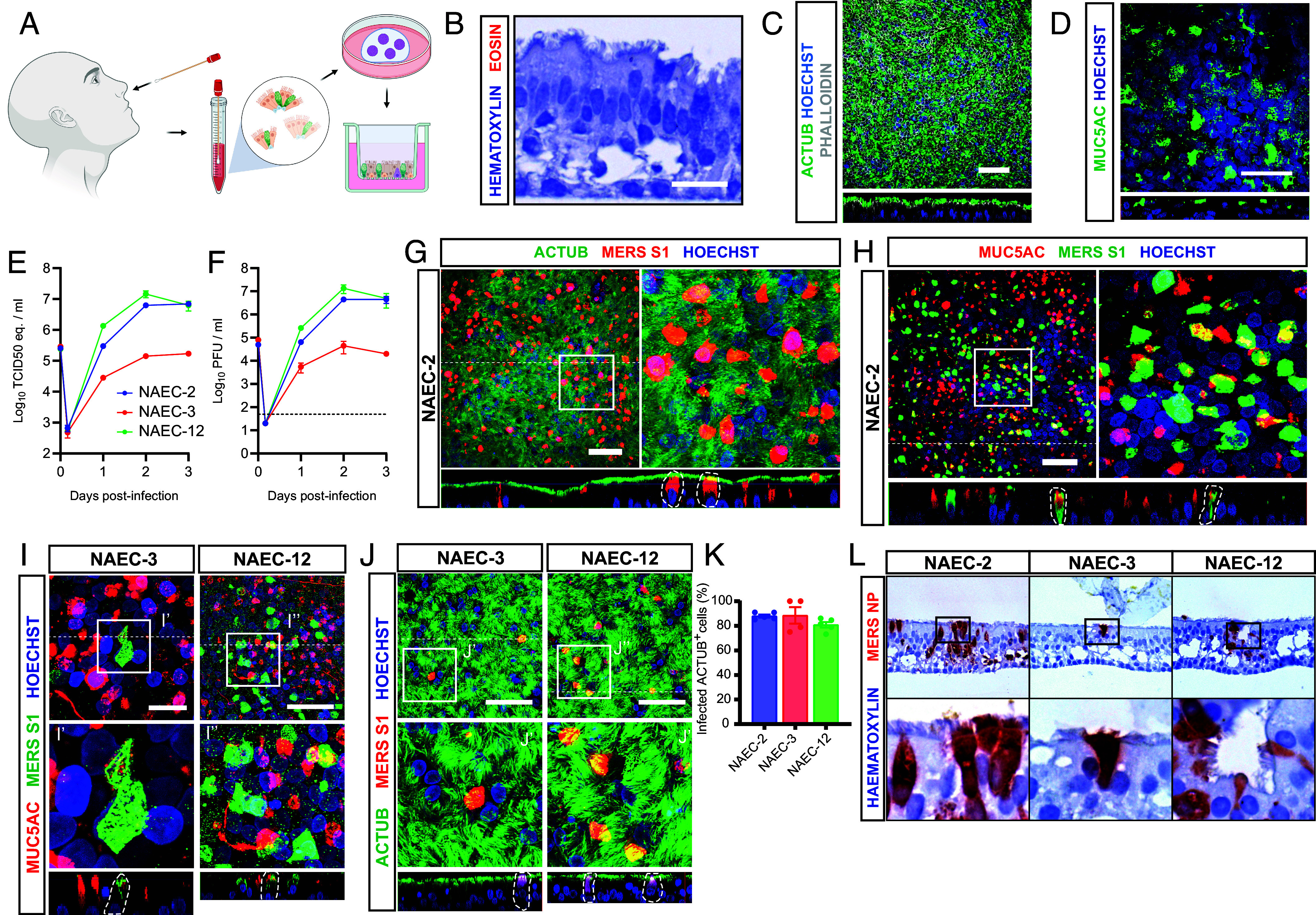
MERS-CoV replicates efficiently in multiciliated cells of human nasal airway cultures. MERS-CoV replication and cellular tropism was determined in human nasal airway organoid-derived epithelial cultures (NAECs). (*A*) Schematic overview of nasal organoid isolation and differentiation. Illustration was created with BioRender.com. (*B*) NAECs were stained with hematoxylin and eosin. (*C* and *D*) NAECs were stained for phalloidin (white) and ACTUB (green, *C*) and MUC5AC (green, *D*). (*E* and *F*) NAECs from three donors (NAEC-2, NAEC-3, and NAEC-12) were infected with MERS-CoV at moi 0.1, and virus was detected in the culture supernatant by RT-qPCR (*E*) and plaque assay titration (*F*). Data are shown as mean and SEM of three biological replicates. The dotted line shows limit of detection. (*G*–*J*) Infected NAECs from three donors were imaged at 3 dpi. (*G*) Infected NAEC-2 were imaged for ACTUB (green) and MERS-S1 (red). (*H*) Infected NAEC-2 were imaged for MUC5AC (red) and MERS-S1 (green). (*I*) Infected NAEC-3 and NAEC-12 were imaged for MUC5AC (red) and MERS-S1 (green). (*J*) Infected NAEC-3 and NAEC-12 were imaged for ACTUB (green) and MERS-S1 (red). *I*’, *I*”, *J*’, and *J*” show digital zoom of the original image. (*K*) Percentage ACTUB^+^-infected cells were quantified at 3 dpi. Data are shown as mean with SEM from four to five replicate images from one infected insert. Each dot represents one replicate. (*L*) IHC for MERS-NP (red) was performed at 20× magnification on FFPE infected NAECs at 3 dpi. Experiments were repeated at least once. Representative images of each staining are shown. Nuclei (blue) were stained with Hoechst. The dotted line indicates the position of the orthogonal view within the z-stack. (Scale bars represent 50 µm.) Inserts (squares) show digital zoom of the original image.

To investigate the tropism of MERS-CoV for the upper respiratory tract we utilized well-differentiated organoid-derived human nasal airway epithelial cultures (NAECs) that were isolated from nasal cytological brushings from healthy adults (Fig. 4*A*), as described previously by other groups (59, 60). Nasal organoids were initially grown in a 3D basement-membrane matrix with an airway organoid medium adapted from Sachs et al. (53, 61). Next, the organoids were differentiated on ALI for at least 6 wk to form NAECs with a pseudostratified respiratory epithelium, presenting multiciliated and secretory cells (Fig. 4 *B*–*D*), similar to the nasal cytological brush materials (*SI Appendix*, Fig. S7*A*). We observed no SCGB1A1^+^ club cells in our NAECs and nasal brush materials, similar to another study (57).

We infected our well-differentiated nasal organoid-derived cultures from three different donors (NAEC-2, NAEC-3, and NAEC-12) with MERS-CoV at a moi of 0.1 (Fig. 4 *E* and *F*). MERS-CoV-infected cultures from NAEC-2 and NAEC-12 efficiently, reaching an infectious viral titer of ~10^7^ pfu/mL at 3 dpi. In NAEC-3, MERS-CoV titers were lower, reaching titers of ~10^4^ pfu/mL (Fig. 4 *E* and *F*). To investigate the tropism of MERS-CoV in the nasal airway cultures we stained infected cells with multiciliated cell markers and secretory cell markers at 3 dpi (Fig. 4 *G* and *H*). In accordance with our earlier data in the pulmonary airway cultures, we observed that MERS-CoV primarily infected multiciliated cells and to a lesser extent goblet cells in NAEC-2 (Fig. 4 *G* and *H*). We confirmed these data using the IF in the other two donors and detected a similar multiciliated cell tropism for MERS-CoV (Fig. 4 *I* and *J*). In line with viral titers, fewer infected cells were detected in NAEC-3 (Fig. 4 *G*–*L*). Tropism was quantified in all donors, showing that ~80% of infected cells were ACTUB^+^ multiciliated cells (Fig. 4*K*). The tropism of multiciliated cells was confirmed using IHC on culture cross-sections (Fig. 4*L*). In conclusion, these data show that MERS-CoV preferentially infects multiciliated cells of the human nasal epithelium with donor variation. However, donor variability was not related to cellular tropism of MERS-CoV.

### MERS-CoV Replication in Nasal Airway Cultures Correlates with DPP4 Expression Levels.

**Fig. 5.**
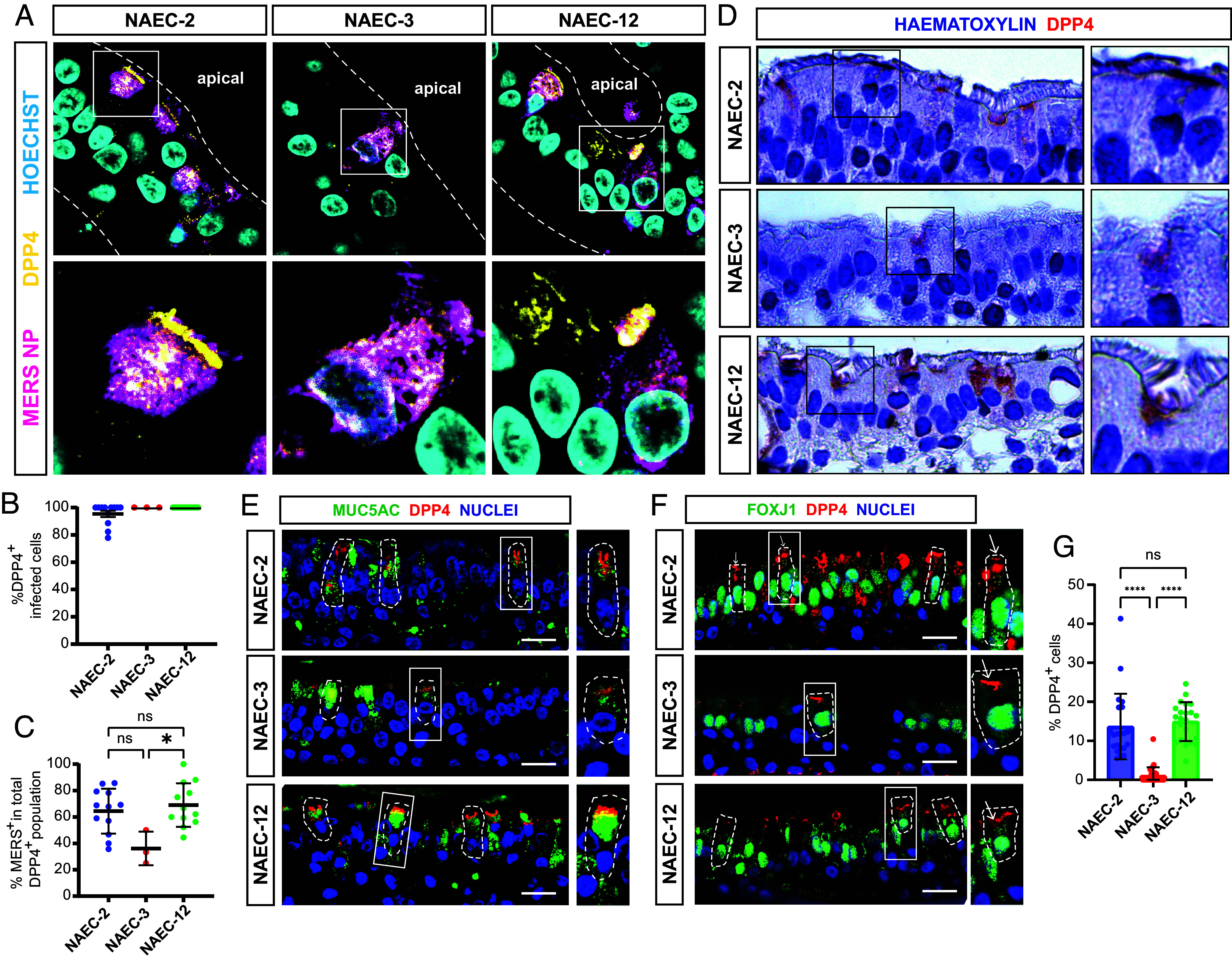
MERS-CoV replication in nasal airway cultures correlates with DPP4 expression levels. Imaging was used to correlate the cellular tropism of MERS-CoV in the NAECs to DPP4 expression. (*A*) NAECs were infected with MERS-CoV, formalin-fixed and paraffin-embedded at 3 dpi and imaged for MERS-NP (magenta) and DPP4 (yellow). Nuclei (blue) were stained with Hoechst. Images were taken at 40× magnification. (*B*) DPP4 expression was quantified in the infected population (MERS-CoV NP^+^) using IF on infected FPPE cultures from NAEC-2, -3, and -12. Ten images of at least two nonsequential tissue sections were used for quantification. Figures show percentage DPP4^+^ cells in the infected cell population. Data are shown as mean and SD. (*C*) Depiction of the same DPP4 quantification data shown as percentage infected cells in the total DPP4^+^ population for each donor. Significance was tested using the nonparametric Kruskal–Wallis with multiple comparisons test. * depicts *P* < 0.05. Infection in NAEC-3 was scarce and could only be detected in three out of 10 images for panels (*B* and *C*). (*D*) IHC was performed for DPP4 (red) on uninfected FFPE NAECs and images were taken at 40× magnification. (*E* and *F*) FFPE cultures from NAEC-2, -3, and -12 were imaged for DPP4 (red) and cell markers (green) MUC5AC (*E*) and FOXJ1 (*F*) using IF. Nuclei (blue) were stained with Hoechst or TO-PRO. (Scale bars represent 20 µm.) (*G*) DPP4 expression data from (*E* and *F*) shown as percentage DPP4^+^ cells in the total cell population. Some images for NAEC-3 did not contain DPP4. Data are shown as mean and SD. Significance was tested using nonparametric Kruskal–Wallis with multiple comparisons test. **** depicts *P* < 0.0001. Experiments were repeated at least once. Representative images of each staining are shown. Inserts (squares) show digital zoom of the original image.

Next, we investigated whether the donor variation in MERS-CoV replication in our NAECs was related to differential DPP4 expression, as previously reported for primary human airway epithelial cultures (62). We performed IF on FFPE MERS-CoV-infected NAECs at 3 dpi (Fig. 5 *A*–*C*). We observed that MERS-CoV^+^ cells coexpressed DPP4^+^ cells (Fig. 5 *A*–*C*). We observed by IHC that DPP4 was expressed variably in ciliated and nonciliated cells across donors (Fig. 5*D*). Isotype control showed no background staining (*SI Appendix*, Fig. S9*A*). DPP4 expression was quantified using IF on FFPE cultures, showing that DPP4 was expressed in MUC5AC^+^ secretory cells and FOXJ1^+^ multiciliated cells (Fig. 5 *E* and *F*). Roughly 10% of cells were MUC5AC^+^/DPP4^+^ and roughly 5% were FOXJ1^+^/DPP4^+^ for NAEC-2 and 12 (*SI Appendix*, Fig. S8 *A* and *C*), while less than 2% of cells were DPP4^+^ within both cell populations for NAEC-3 (*SI Appendix*, Fig. S8*B*). Overall, we detected less DPP4^+^ cells in NAEC-3 compared with NAEC-2 and NAEC-12 (Fig. 5*G*), correlating with MERS-CoV replication kinetics (Fig. 4 *E* and *F*). Cell compositions were similar between donors, only SAEC-2 composed of slightly more FOXJ1^+^ cells compared with the other two donors (*SI Appendix*, Fig. S8 *D*–*F*). Overall, these data suggest that MERS-CoV preferentially targets the subset of DPP4^+^ multiciliated cells, even though DPP4^+^ secretory cells were more abundant in the cultures, similar as observed in the pulmonary airway epithelium. Furthermore, we show that variable DPP4 expression between donors correlates with MERS-CoV replication.

### DPP4 Is Expressed Focally and Variably in Multiciliated Cells of the Human Nose.

**Fig. 6.**
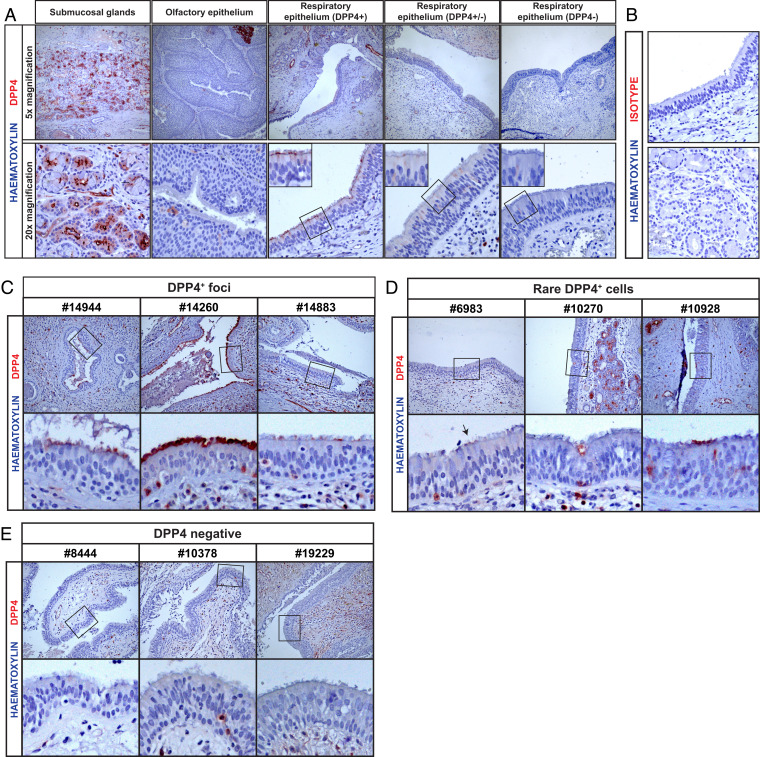
DPP4 is expressed focally and variably in the human nose. Immunohistochemistry for DPP4 was performed on nose tissues from 10 donors: #2551, #14944, #14260, #14883, #6983, #10270, #10928, #8444, #10378, and #19229. (*A*) Various areas are shown from one donor #2551 at 5× and 20× magnification. Foci of respiratory epithelium that expressed DPP4 were indicated (+), foci with faint expression (±) and no expression (−). (*B*) Isotype control staining of respiratory epithelium and submucosal glands from donor #2551. (*C*–*E*) The respiratory epithelium is shown for (*C*) donors with DPP4^+^ foci, (*D*) donors with rare DPP4^+^ cells, and (*E*) DPP4-negative donors. Representative images of each staining are shown. Inserts (squares) show digital zoom of the original image.

Since other studies had not observed DPP4 expression in the human nose (41, 42), we characterized the DPP4 expression in paraffin-embedded nasal tissues from 10 donors (Fig. 6). In one nasal tissue donor, we detected efficient DPP4 expression in the nasal submucosal glands but not in the olfactory epithelium and the majority of the respiratory epithelium (Fig. 6*A*). However, we observed foci of DPP4^+^ cells in the ciliated respiratory epithelium (Fig. 6*A*), indicating that some nasal multiciliated cells can express DPP4, similar to our nasal organoid-derived cultures. We also observed foci with faint DPP4 expression in the same donor (Fig. 6*A*). Isotype control showed no background staining (Fig. 6*B*). These data indicate that DPP4 is expressed in certain regions of the ciliated nasal respiratory epithelium. Next, we characterized the DPP4 expression in nasal tissue from nine more donors to investigate whether DPP4^+^ foci within the respiratory epithelium could be detected across all donors (Fig. 6 *C*–*E*). We observed foci with DPP4^+^ cells in three other nasal tissue donors (Fig. 6*C*). In contrast, rare DPP4^+^ cells were detected in three more donors while most respiratory cells were negative, whereas the respiratory epithelium was completely negative in another three donors (Fig. 6 *D* and *E*). Isotype control showed no background staining in these donors (*SI Appendix*, Fig. S9*B*). Sampling variation and clinical background should be considered when interpreting these results (*SI Appendix*, Table S2), as this might have affected the detection of DPP4. Taken together, in four out of 10 donors foci were observed with DPP4^+^ cells. Overall, our data demonstrate that DPP4 is expressed variably and focally in the respiratory epithelium of human nasal tissues and that DPP4 is detected on the multiciliated cells in these tissues.

## Discussion

Multiciliated cells in the upper respiratory tract are a prime target of respiratory viruses with efficient human-to-human transmission. However, whether MERS-CoV could efficiently target these cells remained ambiguous. In this study, we showed by utilizing scRNA-seq, IF, and IHC that MERS-CoV preferentially infected the multiciliated cells of well-differentiated organoid-derived pulmonary and nasal airway cultures. Furthermore, we demonstrated that DPP4 was expressed variably and focally in human nasal tissues, correlating with DPP4 expression in a subset of multiciliated cells in airway cultures. Taken together, our research suggests that MERS-CoV may effectively replicate in the nasal epithelium of some individuals.

To date, MERS-CoV tropism in humans has mainly been studied in ex vivo and in primary cell cultures (35, 41, 62–65). In these studies, MERS-CoV antigen was mainly detected in nonciliated cells, but the exact target cells were poorly defined. Here, we have shown that MERS-CoV can target nonciliated cells, specifically MUC5AC^+^ and SCGB1A1^+^ secretory cells, similar to previous reports (35, 41, 62–65). However, we showed that 75 to 80% of MERS-CoV-infected cells were multiciliated cells in our well-differentiated LAECs, SAECs, and NAECs, based on IF and IHC. We demonstrated by scRNA-seq in SAECs that mature multiciliated cells comprised ~15% of infected cells, and ~55% of MERS-CoV^+^ cells clustered together in an “infected” population that likely included cells from the ciliated lineage due to the expression of multiciliated cell markers. Together, infected cells of the ciliated clusters and “infected” cluster accounted for ~70% of infected cells in the scRNA-seq dataset, similar as the infected ciliated population that was observed with IF. The clustering of the “infected” population in our scRNA-seq dataset could be explained by the downregulation of various marker genes as a result of coronavirus infections (66–68), and the mRNA overload of MERS-CoV-related transcripts in infected cells. A similar clustering of infected cells has been observed in SARS-CoV-2-infected nasal cells of COVID-19 patients (31). Alternatively, the “infected” cells could be multiciliated cells in a stage of development or in a stage of dedifferentiation as shown for SARS-CoV-2 (66). Multiple populations of developing ciliated cells have been identified (56, 57), including the SCGB1A1^+^ club/ciliated cells that progressively express multiciliated markers during their differentiation, but whether these cell types are specifically targeted by MERS-CoV remains to be determined. We observed a minor population of epithelial cells in our cultures expressing DPP4, consistent with previous findings (62). Interestingly, the abundance of DPP4^+^ secretory cells was higher than DPP4^+^ multiciliated cells in our well-differentiated pulmonary and nasal cultures. These data indicate that DPP4 expression cannot fully explain the preferential multiciliated tropism of MERS-CoV. Other factors, such as the transmembrane protease serine 2 (TMPRSS2) which is most abundantly expressed in multiciliated cells of human airway tissues (31, 69), may also contribute to the infection of these cells in consort with DPP4. Alternatively, cilia motility might increase the infection of multiciliated cells by MERS-CoV when its entry factors are expressed, as observed for SARS-CoV-2 (70). Further delineating the factors that determine the preferential tropism of MERS-CoV for multiciliated cells should be considered in follow-up studies. Infection of multiciliated cells by MERS-CoV has been observed by others (35, 62), but in contrast to our data, this was always considered a minor population. Shorter differentiation of airway cultures might partially explain these results, as we showed that this skewed the MERS-CoV tropism toward secretory cells in a DPP4-dependent manner. Loss of ciliary coverage and downregulation of FOXJ1 might further contribute to the nonciliated cell tropism of MERS-CoV observed in other studies. We observed a reduction of infected multiciliated cells at later stages of infection, consistent with findings from a previous study (62). This reduction may be attributed to infected cell shedding or death, as well as loss of ciliary coverage. Although the exact fate of MERS-CoV-infected cells remains largely unknown, especially during later stages of infection, we did not observe clear cytopathic effects as result of MERS-CoV replication, whereas severe cytopathology was observed for human metapneumovirus using similar cultures (71). Loss of ciliary coverage has been demonstrated in the respiratory tissues of MERS-CoV-infected animals (33, 72), and denuding of the bronchiolar epithelium has been observed in an autopsy report of a fatal MERS-CoV case (73). Significant cell shedding and loss of ciliated cells have also been shown for SARS-CoV (74) and SARS-CoV-2 (66, 75), where widespread infection caused dedifferentiation of the airway epithelium by downregulation of FOXJ1 (66). Interestingly, motile cilia are essential for efficient virus replication of SARS-CoV-2 in multiciliated cells (70), indicating that ciliopathy might hamper coronavirus dissemination in the respiratory tract, however, whether similar mechanisms are present for MERS-CoV remains to be determined.

There is a general consensus that MERS-CoV is an upper respiratory tract pathogen in dromedary camels and a lower respiratory tract pathogen in humans due to the tissue variation in DPP4 expression (41, 42). The upper respiratory tract tropism of MERS-CoV in humans remained ambiguous, regardless of various indications that cryptic community transmission and subclinical infections occur (10, 27, 28). In this study, we demonstrated that MERS-CoV productively replicates in NAECs. Few studies show in vitro MERS-CoV replication in primary human nasal cell cultures (63, 76), either by utilizing cells from individual donors (63) or pooled donors (76). Donor differences were observed by Otter et al. (63). These authors showed that MERS-CoV replicated in two out of six nasal cell donors. We demonstrate that MERS-CoV can replicate in organoid-derived NAECs from three donors, albeit to differential peak titers. We show that these donor differences were likely attributed to variable DPP4 expression. More abundant DPP4 expression was associated with higher MERS-CoV titers. On the other hand, other factors that may determine donor variation, such as variable antiviral innate immune responses, cannot be ruled out. We presented evidence of DPP4^+^ foci in the human nasal respiratory epithelium of four out of 10 nasal tissue donors, showing a varied DPP4 distribution within the human nasal epithelium and between individuals. In these foci, DPP4 was presented on multiciliated cells, correlating to the preferential tropism of MERS-CoV for multiciliated cells in the NAECs. These data augment earlier studies which show that DPP4 expression is subject to extensive local regulation and interindividual variation (40, 62, 63, 77, 78) and that DPP4 expression in human airway epithelial cells positively correlates to MERS-CoV replicative titers (62). Interestingly, Otter et al. (63) showed that MERS-CoV replicated to infectious virus peak titers of ~10^5^ at 6 dpi in the human primary nasal cells when infected with a moi of 5. MERS-CoV replication in our organoid-derived NAECs was more efficient, reaching infectious virus peak titers of up to ~10^7^ at 3 dpi when infected with a moi of 0.1, suggesting that the NAECs used in our study were more susceptible to MERS-CoV. This variation in replication efficiency might have been related to the nonciliated cell tropism of MERS-CoV and limited DPP4 expression in primary nasal epithelial cell cultures (63). The detection of DPP4 in multiciliated cells of organoid-derived NAECs and human nasal tissues suggests that the tropism of MERS-CoV for multiciliated cells may more accurately reflect the in vivo situation. Taken together, these recent studies indicate that the human nasal epithelium is a likely site for MERS-CoV replication.

The biological relevance of DPP4^+^ foci in the human airway epithelium remains largely unknown. DPP4 is a membrane-bound peptidase with a wide range of substrates, including cytokines, growth factors, and hormones, which it can either activate or inactivate (79). The broad distribution of DPP4 across the parenchyma, epithelium, endothelium, and fibroblasts of the human airways suggests it may play an important role in both lung physiology and pathology (80). It has been demonstrated previously that DPP4 is regulated by various stimuli, such as smoking, diabetes, and inflammation (40, 41, 78, 81). Upregulation of DPP4 likely increases the risk of MERS-CoV infection, as comorbidities correlate with MERS-CoV hospitalized patients (82). On the other hand, underlying genetic factors might also explain the differences in DPP4 expression in the respiratory epithelium between individuals, but this has not been studied in detail. It seems that ACE2 is more uniformly expressed in the upper respiratory tract epithelium (36–38), compared to DPP4. Whether this is associated with transcriptional regulatory networks in these cell types would be highly relevant to investigate in the future.

Our research suggests that DPP4 expression may enable MERS-CoV replication and shedding in the upper respiratory tract of some individuals. However, the overall lack of widespread MERS-CoV human-to-human transmission appears to be regulated by several limiting factors. The proportion of individuals that express DPP4 in their nasal epithelium may be too low to sustain transmission chains, resulting in a low basic reproduction number and secondary attack rates observed during MERS-CoV outbreaks. Alternatively, the lack of uniform expression of DPP4 within the nasal respiratory epithelium might limit sustained MERS-CoV replication and shedding in the upper respiratory tract in susceptible individuals. Additionally, other factors may further regulate MERS-CoV transmission, even in the presence of nasal DPP4^+^ foci, including insufficient α2,3-linked sialic acid expression in the human nose (83), mucin-related infection inhibition similar to that observed in SARS-CoV-2 (84), or reduced viral particle stability in respiratory droplets. While our results provide important insights into the tropism of MERS-CoV in the human pulmonary and nasal airway epithelium, our findings also underscore how little we understand about the determinants for human-to-human transmission of this virus.

The periodical emergence of MERS-CoV and the large nosocomial outbreaks that occurred in the past underscore the significance of investigating MERS-CoV transmission. As replication of MERS-CoV in the human nasal epithelium of some individuals is probable, future research should focus on characterizing the implications and the extent of upper respiratory tract shedding in MERS-CoV-infected individuals, as this is crucial for developing novel antiviral and containment strategies for future outbreak preparedness in the absence of widespread vaccination. Continued exposure of MERS-CoV in humans and replication in the nose can drive viral adaptation to the human upper respiratory tract, potentially leading to the emergence of more transmissible viral variants with pandemic potential in the future. Adaptation of MERS-CoV to the human upper respiratory tract might be associated with an increased likelihood for subclinical infections, possibly resulting in more widespread cryptic transmission and decreased hospitalization, complicating MERS-CoV surveillance. Therefore, it is crucial to continue surveillance and characterization of the currently circulating and newly emerging MERS-CoV variants.

## Materials and Methods

Human adult pulmonary airway organoids were isolated from dissected bronchus ring tissue (large pulmonary airway) or lung parenchyma (small pulmonary airway) obtained from residual nontumor lung tissue of patients undergoing lung resection surgery. The Medical Ethical Committee of the Erasmus MC Rotterdam granted permission for this study (METC 2012-512). Human adult nasal airway organoids were isolated from the nasal cavities of multiple adult donors by cytological brushings after obtaining informed consent. Individuals with a known respiratory disease, an autoimmune disease, or receiving immunosuppressive treatment were excluded from the study. This study protocol was approved by the Medical Ethical Committee of the Erasmus MC Rotterdam (MEC-2022-0164). All donor materials were completely anonymized and nonidentifiable prior to use in this study. The human large and small pulmonary airway and nasal airway organoids were plated on transwell inserts and subsequently differentiated on air–liquid interface for at least 6 wk to form well-differentiated organoid-derived epithelial cultures (LAECs, SAECs, and NAECs). Once fully differentiated, the cultures were characterized for DPP4 and cell marker expression by immunofluorescent staining, and immunohistochemistry. Next, the cultures were infected with MERS-CoV to delineate its cellular tropism in the pulmonary and nasal airways. MERS-CoV replication was determined by measuring virus release in the supernatant by qRT-PCR and plaque assay titration. MERS-CoV tropism was determined by single-cell RNA sequencing, immunofluorescent staining, and immunohistochemistry. To characterize the DPP4 expression distribution in the human nasal mucosa by immunohistochemistry, FFPE human nose tissues were acquired from the historical patient tissue database of the Department of Pathology, Erasmus MC Rotterdam. These tissues consisted of surplus healthy tissue adjacent to the surgical site, collected from patients undergoing nasal surgery for various clinical reasons, and included both biopsy and resection materials. All of the techniques that are used in this manuscript are described in more detail in *SI Appendix*, *Materials and Methods*. All materials or protocols mentioned in this work can be obtained by contacting the corresponding author.

## Supplementary Material

Appendix 01 (PDF)

## Data Availability

All study data are included in the article and/or *SI Appendix*. The single-cell mRNA sequencing dataset has been deposited in the Gene Expression Omnibus database (GSE289955) (85).
