# Supplementary material for: Variable DPP4 expression in multiciliated cells of the human nasal epithelium as a determinant for MERS-CoV tropism
Source: Proc Natl Acad Sci U S A. 2025 Mar 6;122(11):e2410630122. doi: 10.1073/pnas.2410630122 (PMC11929475; doi:10.1073/pnas.2410630122)
Supplement: Supplementary file 1 — Appendix 01 (PDF) [file pnas.2410630122.sapp.pdf]

Supporting Information for

# Variable DPP4 expression in multiciliated cells of the human nasal epithelium as a determinant for MERS-CoV tropism

Tim I. Breugem[a], Samra Riesebosch[a,e], Jingshu Zhang[a], Anna Z. Mykytyn[a], Lisette Krabbendam[b,f], Nathalie Groen[c], Sivana Baptista Varela[a], Debby Schipper[a], Petra B. van den Doel[a], Romy van Acker[a], Ralph Stadhouders[b], Mart M. Lamers[a,d,1], Bart L. Haagmans[a,1]*

*Corresponding author: Bart L. Haagmans

**Email:** b.haagmans@erasmusmc.nl (B.L.H.)

**This PDF file includes:**

Materials and Methods
Tables S1 to S2
Figures S1 to S9
SI References

## Supporting Information

## Materials and Methods

**Cell lines.** Calu-3 cells (ATCC HTB-55) were maintained in Opti-MEM I (1×) + GlutaMAX (Gibco) supplemented with 10% fetal bovine serum (FBS), penicillin (100 IU/mL), and streptomycin (100 IU/mL). Vero cells (ATCC) were maintained in Dulbecco's modified Eagle's medium (DMEM, Gibco) supplemented with 10% fetal bovine serum (FBS), HEPES (20 mM, Lonza), sodium pyruvate (1 mM, Gibco), penicillin (100 IU/mL), and streptomycin (100 IU/mL). Both cell lines were kept at 37°C in a humidified CO2 incubator and were tested negative for mycoplasma.

**Isolation and culture of human pulmonary airway organoids.** Adult human pulmonary tissue was obtained from residual non-tumor lung or bronchus tissue of patients undergoing lung resection surgery for lung cancer. The Medical Ethical Committee of the Erasmus MC Rotterdam granted permission for this study (METC 2012-512). All donor materials were completely anonymized and non-identifiable prior to use in this study. The available donor details were provided in table S2. Human adult stem cells were isolated from dissected bronchus ring tissue (large pulmonary airway) or lung parenchyma (small pulmonary airway), containing bronchiolar and distal lung tissue, and maintained as described before using a protocol adapted from Sachs and colleagues (1, 2). Tissues were cut into ~4 mm sections, washed three times in 1 mL Advanced DMEM/F12 (Gibco) with Glutamax (1× Gibco), HEPES (10 mM Lonza), and primocin (50 µg/ml, nvitrogen) (AdDF+++), and incubated with 1mL  dispase (Corning) for 1 hour at 37˚C. The digested tissues were mechanically sheared using pipetting to release stem cells from the tissues. If stem cells were not released, incubation and shearing steps were repeated with 15 min intervals. Next, digested tissues were filtered with 100 µm strainer (Corning) and washed 3 times using 5 mL AdDF+++ to remove any residual dispase. If blood platelets were present in the pellets, these were removed using 5 min incubation with 500 µL red blood cell lysis buffer (Roche) and 3 times washing with 5 mL AdDF+++ to remove residual lysis buffer. After each washing or incubation step cells were centrifuged at 400 × g for 2 min. Stem cells were plated in 30-40 µL BME type-II RGF Select (Biotechne) droplets in 300 µL AO medium in 48 well suspension culture plates to form 3D human pulmonary airway organoids. Organoids were kept at 37°C in a humidified CO2 incubator. Medium was replaced every 4 to 5 days. Organoids were split into ~1:5 fractional ratios using TrypLE digestion, depending on cell density. Organoid cultures were tested negative for mycoplasma.

**Isolation and culture of human nasal airway organoids.** Human nasal airway stem cells were isolated from the nasal cavities of multiple adult donors by cytological brushings after obtaining informed consent, as previously described by other groups (3, 4). Individuals with a known respiratory disease, an autoimmune disease, or receiving immunosuppressive treatment were

excluded from the study. The study protocol was approved by the Medical Ethical Committee of the Erasmus MC Rotterdam (MEC-2022-0164). All donor materials were completely anonymized and non-identifiable prior to use in this study. The available donor details were provided in table S2. After the nasal cavity brushings were acquired they were kept in AdDF+++ on ice until use. The epithelial tissues were detached by washing the brushes with AdDF+++. The brush material was either directly formalin-fixed for subsequent use or digested using 1 mL TrypIE to dissociate the stem cells, washed three times in 5 mL AdDF+++ and plated in 30-40 µL BME type-II (Biotechne) droplets, similar to processing human pulmonary airway organoids. If blood platelets were present in the cultures, these were removed using 5 min incubation with 500 µL red blood cell lysis buffer (Roche) and 3 times washing with 5 mL AdDF+++ to remove residual lysis buffer. After each washing or incubation step cells were centrifuged at 400 × g for 2 min. The stem cells were plated in 30-40 µL BME type-II RGF Select (Biotechne) droplets in 300 µL $AO^{ENC}$ medium in 48 well suspension culture plates (Greiner) to form 3D nasal airway organoids. AO was supplemented with EGF (50 ng/mL, peprotech), CHIRR99021 (3 µM , Stemcell), and NRG-1 (100 ng/mL, Peprotech) ($AO^{ENC}$). Organoids were kept at 37°C in a humidified CO2 incubator. The organoids were split with 1:2 to 1:4 fractional ratios using TrypIE digestion. Organoid cultures were tested negative for mycoplasma.

**Differentiation of pulmonary and nasal airway organoids at the air-liquid interface.** 3D pulmonary or nasal airway organoids were harvested by mechanical disruption of the BME droplets in 500 µL ice-cold AdDF+++. Residual BME and cell debris were removed by washing 3 times in 5 mL ice-cold PBS. Organoids were digested with 1 mL TrypLE for 5-10 min and mechanically disrupted to single-cell suspension using pipetting. TrypLE was neutralized with 1 mL FBS for 30 seconds. The cells were subsequently washed 3 times with 5 mL AdDF+++ to remove residual TrypLE and FBS. The single cells were plated on transwell inserts (CELLTREAT, 0.4 µm pore Polyethylane membrane inserts, 24-well format) using commercially available Pneumacult-ALI medium (complete base medium with 1× maintenance supplement; Stemcell) and AO (pulmonary airway) or $AO^{ENC}$ (nasal airway) in 1:1 ratio to form monolayers. 200 µL medium was added to the top compartment and 500 µL to the bottom compartment. Transwell inserts were coated prior to plating with rat tail collagen type I (250 µg/ml, Thermo) in 200 µL AdDF+++ for 1 hour at 37 degrees. Roughly 50.000 cells were plated per well for pulmonary airway cultures and 200.000 cells for nasal airway cultures. When the monolayer had formed and grown to 100% confluency, generally after 24 to 48 hours, the medium was removed from the top compartment and the medium was replaced in the bottom compartment with 500 µL Pneumacult-ALI medium to form an air-liquid interface (ALI). Medium was replaced and cells were washed at the top compartment every 4 to 5 days. Cells were differentiated at ALI for at least 6 weeks, except when stated otherwise, to obtain well-differentiated organoid-derived cultures with an 3D pseudostratified epithelium.

Commercially available human primary airway SmallAir™ cultures (Epithelix) were obtained from micro-dissection of the bronchioles of human lung samples from healthy non-smokers, under ethical approval and donor consent. SmallAir™ cultures were differentiated at ALI for at least 6 weeks in 500 µL SmallAir™ Culture Medium before use in infection experiments. Medium was replaced and cells were washed with 200 µL AdDF+++ at the top compartment every 4 to 5 days.

**Infection of airway organoid-derived epithelial cultures.** Infection of human ALI cultures was performed as described previously (2, 5). Infection experiments were performed with 6-week differentiated organoid-derived SAECs, LAECs, NAECs or SmallAir™ primary cell cultures. Infection experiments were conducted with three biological replicate cultures. Prior to infection, the ALI monolayers were washed three times with 200 µL AdDF+++ for 10 min at 37˚C in a humidified CO2 incubator, and subsequent pipetting, to remove the secreted mucus layer. The monolayers were subsequently infected with MERS-CoV at the indicated multiplicity of infection (moi) in 200 µL AdDF+++ and incubated at 37°C with 5% CO2 for 2 h before washing three times in 200µL AdDF+++ to remove inoculum. A sample of 200 µL excess inoculum was stored at -80°C prior to infection for titration. Cells were incubated at 37°C with 5% CO2 until virus sample timepoints were taken. To harvest virus, the monolayers were incubated apically with 200µL AdDF+++ at 37°C with 5% CO2 for 10 min at indicated time points post infection. The medium was aspirated apically (supernatant) by pipetting 10 times, and was stored at -80°C. The samples at day 0 were taken directly after washing. All samples were thawed at centrifuged at 2,000 × g for 5 min, and virus titers were determined in the supernatant by qRT-PCR (RNA) and plaque assay titration (live virus). All work with infectious MERS-CoV was performed in a Class II Biosafety Cabinet under BSL-3 conditions at Erasmus MC Rotterdam.

**MERS-CoV propagation and titration.** All MERS-CoV infection experiments were performed with MERS-CoV/EMC isolate (HCoV-EMC/2012; NC_019843.3), and viral stocks were produced as described previously (6). Virus from early passage was used to infect Vero cells medium at a moi of 0.01 in Advanced DMEM/F12 (Gibco), supplemented with HEPES (20 mM), 1× Glutamax, penicillin (100 IU/mL) and streptomycin (100 IU/mL) and incubated at 37°C in a humidified CO2 incubator. The supernatant was harvested at 72h post infection and cleared by centrifugation at 1,000 × g for 5 min and stored at -80°C degrees in aliquots. Virus stocks were deep sequenced and were confirmed to be genetically identical to the original material and used for subsequent experiments. All work with infectious MERS-CoV was performed in a Class II Biosafety Cabinet under BSL-3 conditions at Erasmus Medical Center.

Viral stocks and experiment samples were titrated by a plaque assay on Calu-3 cells as reported previously (7). In short, viral stocks or experiment samples were thawed, spun down at 1000 × g

for 5 min and serially diluted in 10-fold dilutions in 200 ul Opti-MEM (1×) with GlutaMAX (1×). 100 ul of each dilution were added to monolayers of 5 × 105 Calu-3 cells in the same medium in a 24-well plate. Cells were incubated with inoculums at 37°C for 2 h, and then medium was replaced with 1.2% Avicel (FMC biopolymers) in Opti-MEM I (1×) with GlutaMAX (1×) for 2 days. Next, cells were fixed in 4% formalin for 20 min, permeabilized in 70% ice-cold ethanol, and washed in PBS. Cells were blocked in 0.6% BSA (bovine serum albumin; Sigma) in PBS and stained primary antibodies specific for MERS-CoV antigens (Table 1) in PBS containing 0.3% BSA, washed three times in PBS, and stained with corresponding secondary antibodies (Table 1) in PBS containing 0.3% BSA. Cells were washed twice in PBS, and plates were scanned with the Amersham Typhoon Biomolecular Imager (channel Cy2; resolution 10 μm; GE Healthcare). All staining steps were performed at room temperature for 30 min. Plaque assay analysis was performed using ImageQuant TL 8.2 software (GE Healthcare).

**Determination of virus titers using qRT-PCR.** MERS-CoV RNA was extracted as described previously for SARS-CoV-2 experiments (5), and RNA genome copies (E-gene) were determined by qRT-PCR. Briefly, supernatant samples were thawed and centrifuged at 2,000 × g (supernatant) for 3 min. A sample of 60 μL was lysed in 90 μL MagnaPure LC Lysis buffer (Roche) at RT for 10 min. RNA was extracted by incubating samples with 50 μL Agencourt AMPure XP beads (Beckman Coulter) for 15 min at room temperature, washing beads twice with 70% ethanol on a DynaMag-96 magnet (Invitrogen) and eluting in 30 μL DEPC-treated water. qRT-PCR using primers targeting the E gene using the following primers (Eurogentec): MERS_E_forward GCAACGCGCGATTCAGT, MERS_E_reverse GCCTCTACACGGGACCCATA and MERS_E_probe CTCTTCACATAATCGCCCCGAGCTCG using 6FAM-BHQ1 labeled fluorescent probe. Viral titers (TCID50 equivalents per mL) were determined by comparing the Ct values to a standard curve derived from a virus stock titrated on Vero cells.

**Fixed immunofluorescence microscopy.** Immunostaining of transwell inserts was performed as described previously (2, 5, 7). In short, transwell inserts or nasal brush material were fixed in 4% paraformaldehyde for 20 min, permeabilized in 70% ethanol, and blocked for 60 min in 10% normal goat serum in PBS (blocking buffer). Cells were incubated with primary antibodies (Table S1) overnight at 4°C in blocking buffer, washed twice with PBS, incubated with corresponding secondary antibodies (Table S1) in blocking buffer for 2 h at room temperature, washed two times with PBS, incubated with Hoechst (thermofisher), washed twice with PBS, and mounted in Prolong Antifade (Invitrogen) mounting medium. Samples were imaged on a LSM700 confocal microscope using ZEN software (Zeiss).

**Quantification of fluorescent imaging.** Quantifications were performed using an LSM700 confocal microscope and ZEN software (Zeiss) or CellProfiler software (8). To quantify the tropism of MERS-CoV, the percentages of MERS-CoV$^+$ cells which co-localized with ACTUB, SCGB1A1 (if applicable) or MUC5AC out of all MERS-CoV$^+$ cells were determined (N=5 z-stack images per group from the same insert, top views, objectification 40x). To compare cilia length of infected cells to uninfected cells in small and large airway cultures, cilia length was measured (N=10 randomly selected cells from at least 2 non-sequential tissue sections, technical replicates of three cilia were used per cell, z-stack cross-sections, objectification 40x). FOXJ1 fluorescent intensity was quantified using ZEN software in the nuclei of MERS-CoV-infected and non-infected cells in human small and large airway cultures. Average fluorescent intensity in nuclei was calculated using ZEN profile analysis in a representative z-stack plane (N=20 cells for 2 biological replicates, representative data shown for one replicate, objectification 40x). Total DPP4$^+$ cells and percentage DPP4$^+$ cells per cell type were quantified in infected or control FFPE organoid-derived cultures imaged for DPP4 and cell markers MUC5AC (goblet cells), SCGB1A1 (club cells), FOXJ1 (multiciliated cells) or MERS-CoV NP (infected cells) (N=10 replicate images of various regions of at least 2 non-sequential tissue sections, maximum intensity projection of four z-stack images or single plane, objectification 40x). When the DPP4 signal could not be allocated to a specific cell, this was excluded from the quantification. The total nuclei per image were quantified using CellProfiler and was used to calculate total cells per image (8). The same imaging data was used to calculate relative cell type abundance based on marker expression in the different organoid-derived culture systems.

**Statistical analysis.** Statistical analysis was performed using Graphpad Prism v10.1.2 software. Prior to statistical test, datasets were tested for normality. Datasets were subsequently analyzed using parametric unpaired students t-test using Welch's correction or nonparametric Mann Whitney U test. Kruskal-Wallis test with multiple comparisons was performed for comparing multiple groups**.** The specific statistical testing is indicated in the figure legend.

**Human nose tissue.** FFPE human nose tissues were acquired from the historical patient tissue database of the Department of Pathology, Erasmus MC Rotterdam. These tissue consisted of surplus healthy tissue adjacent to the surgical site, collected from patients undergoing nasal surgery for various clinical reasons, and included both biopsy and resection materials. Nasal tissues that did not contain respiratory epithelium were excluded from the study. The study protocol was consistent with international ethical and professional guidelines. The use of anonymized rest material is regulated under the code for proper secondary use of human tissue in the Netherlands. The available donor details were provided in table S2.

**Paraffin embedded immunohistochemistry.** Staining was performed on 3 µm thick sections. Slides were deparaffinized by 4 minute incubation in xylene (2x), followed by consecutive 2-minute incubations in 100% ethanol (2x), 96% ethanol, 90% ethanol, 70% ethanol and finally 5 min in PBS. Antigen was retrieved in a boiling citrate buffer (10 mM, pH 6) for 15 min in a microwave at 600 W. Endogenous peroxidase was blocked in 3% H2O2 for 10 min and washed three times with PBS. Cells were blocked with 10% Normal Rabbit Serum or Normal Goat Serum for 30 min, incubated with primary antibody (Table S1) in 0,1% BSA at room temperature for 1 hour or at 4℃ overnight, washed three times with PBS, incubated with HRP labeled secondary antibody in 0,1% BSA for 1 hour, washed three times with PBS, incubated in AEC for 10 min, washed in PBS, counterstained for 5 seconds in hematoxylin, washed in tap water and mounted with Kaiser's Glycerol (Merck Millipore). Isotype controls were taken along with all IHC experiments. Samples were imaged using a light microscope using ZEN software (Zeiss).

**Single-cell RNA sequencing.** Human SAECs from one donor were infected with MERS-CoV at a moi 1. At 1 dpi cells were harvested and re-suspended into a single cell solution using TryplE digestion. The single cells were subsequently fixed using methanol fixation. Single-cell mRNA sequencing was performed at Single Cell Discoveries according to standard 10× Genomics 3' V3.1 chemistry protocol. In short, the single-cell suspension was methanol fixed in 80% methanol and frozen at −80°C. Before loading the cells on the 10× Chromium controller, cells were rehydrated in a rehydration buffer and counted to assess cell concentration. Cells were loaded, and the sequencing library was prepared following the standard 10× Genomics protocol. The DNA library was paired-end sequenced on an Illumina NovaSeq S4 with a 2 × 150 bp Illumina kit.

**Mapping of single-cell RNA sequencing data.** BCL files resulting from sequencing were transformed to FASTQ files with 10× Genomics Cell Ranger mkfastq. FASTQ files were mapped with Cell Ranger count. Read 1 was assigned 150 base pairs during sequencing, but only the first 28 bp were used to identify the Illumina library barcode, cell barcode, and unique molecular identifier (UMI). R2 was used to map to the human # genome GRCh38 supplemented with the sequence for MERS-CoV.

**Clustering and differential gene expression analysis.** The data from all samples were loaded in R (version 4.2.0) and processed using the Seurat package (version 4.1.1). High-quality cells with at least 1000 UMIs/cell and mitochondrial faction below 10% were selected, yielding 2751 cells. The gene counts were normalized for sequencing depth per cell and log-transformed using a scaling factor 10,000. The 2000 most variable genes were scaled and used for graph-based clustering of the cells, followed by the original Louvain algorithm for modularity optimization. The differentially expressed genes per cluster were calculated using the Wilcoxon rank sum test and

used to identify cell types. Clusters belonging to similar cell types were merged and annotated at 3 different levels.

## Tables S1 to S2

**Table S1:** Antibodies used for immunofluorescence and immunohistochemistry.

| ANTIBODY | IDENTIFIER | SOURCE | DILUTION |
|---|---|---|---|
| Goat-anti-DPP4 | AF1180 | R&D systems | 1:50 |
| Goat-anti-SCGB3A2 | AF3545 | R&D systems | 1:100 |
| Mouse-anti-ACTUB | sc-23950 AF488 6-11B-1 | Santa Cruz Biotechnology | 1:100 |
| Mouse-anti-CC10 (SCGB1A1) | sc-390313 AF594 | Santa Cruz Biotechnology | 1:100 |
| Mouse-anti-SCGB1A1 | 10808-MM06 | Sino Biological | 1:1000 |
| Mouse-anti-FOXJ1 | 14-9965-82 | Invitrogen | 1:100 |
| Mouse-anti-MERS-CoV NP | 40068-MM10 | Sino Biological | 1:1000 |
| Mouse-anti-MUC5AC | MA5-12178 45M1 | Invitrogen | 1:100 |
| Mouse-anti-P63 | ab735 | Abcam | 1:100 |
| Mouse-anti-SCGB3A1 | MAB27901 | R&D systems | 1:100 |
| Goat-anti-UGRP1/SCGB3A2 | AF3545 | R&D systems | 1:100 |
| Mouse-anti-SYP | SC-17750 AF594 | Santa Cruz Biotechnology | 1:100 |
| Rabbit-anti-MERS-CoV S | 40069-T52 | Sino Biological | 1:1000 |
| Alexa Fluor 488 goat anti-mouse IgG (H+L) | A11029 | Invitrogen | 1:400 |
| Alexa Fluor 488 goat anti-mouse IgG2a (y2a) | A21131 | Invitrogen | 1:400 |
| Alexa Fluor 488 goat anti-rabbit IgG | ab150077 | Abcam | 1:400 |
| Alexa Fluor 488 rabbit anti-goat IgG (H+L) | A11078 | Invitrogen | 1:400 |
| Alexa Fluor 488 rabbit anti-mouse IgG | ab169345 | Abcam | 1:400 |
| Alexa Fluor 594 goat anti-mouse IgG1 (y1) | A21125 | Invitrogen | 1:400 |
| Alexa Fluor 594 goat anti-mouse IgG1 (y1) | A21125 | Invitrogen | 1:400 |
| Alexa Fluor 594 goat anti-rabbit IgG (H+L) | A11012 | Invitrogen | 1:400 |
| Alexa Fluor 594 rabbit anti-goat IgG (H+L) | A11080 | Invitrogen | 1:400 |
| Alexa Fluor Plus 488 goat anti-rabbit IgG (H+L) | A32731 | Invitrogen | 1:400 |
| Goat-anti-mouse IgG-HRP | P0447 | DAKO | 1:400 |
| Rabbit-anti-goat IgG-HRP | P0160 | DAKO | 1:400 |

**Table S2.** Background information from the human tissue donors used in this study.

| Donor | Identifier | Sex | Age | Sample origin | Smoker | COPD | Clinical background and other comorbidities | Used in figure |
|---|---|---|---|---|---|---|---|---|
| Lung tissue donor 1 | LAEC-1 and SAEC-1 | Male | 74 | Non-tumor dissected bronchus and distal lung resection | Former | Unknown | Lung tumor (adenosquamous carcinoma) | Fig. 1-2 and S1-6 |
| Lung tissue donor 2 | SAEC-2 | Female | 67 | Non-tumor distal lung resection | Yes | Unknown | Lung tumor (undifferentiated squamous cell carcinoma) | Fig. 3 |
| Lung tissue donor 3 | SAEC-3 | Male | 58 | Non-tumor distal lung resection | Former | Unknown | Lung tumor (papillary-predominant adenocarcinoma) | Fig. 3 |
| Commercial airway culture donor 1 | CAE-1 | Unknown* | Unknown* | Micro-dissection of the bronchioles from healthy human lung | No | Unknown* | Unknown* | Fig. 3 |
| Commercial airway culture donor 2 | CAE-2 | Unknown* | Unknown* | Micro-dissection of the bronchioles from healthy human lung | No | Unknown* | Unknown* | Fig. 3 |
| Nose brush donor 2 | NAEC-2 | Male | 24 | Cytological brushing of nasal cavity | No | Unknown | Unknown | Fig. 4-5 and S8 |
| Nose brush donor 3 | NAEC-3 | Female | 26 | Cytological brushing of nasal cavity | No | Unknown | Unknown | Fig. 4-5 and S8 |
| Nose brush donor 12 | NAEC-12 | Male | 31 | Cytological brushing of nasal cavity | No | Unknown | Unknown | Fig. 4-5 and S8 |
| Nose brush donor 31 | NAEC-31 | Male | 21 | Cytological brushing of nasal cavity | Unknown | Unknown | Unknown | Fig. S7 |
| Nose tissue donor 1 | 2551 | Male | 49 | Nasal sinus (right) | Yes | No | Nasal inverted papilloma‡, obstructive Sleep Apnea Syndrome | Fig. 6 and S9 |
| Nose tissue donor 2 | 14944 | Female | 61 | Nasal sinus (left) | No | No | Nasal inflammation with eosinophilic infiltrate‡, asthma | Fig. 6 and S9 |
| Nose tissue donor 3 | 14260 | Male | 76 | Nasal cavity | Former | Yes | Nasal IgG4 related disease‡, benign prostate hyperplasia | Fig. 6 and S9 |
| Nose tissue donor 4 | 14883 | Female | 43 | Nasal sinus (left) | No | No | Nasal fibrosis and granulosis‡, gastric bypass, viral meningitis | Fig. 6 and S9 |
| Nose tissue donor 5 | 6983 | Female | 67 | Nasal sinus (right) | No | No | Nasal cystous carcinoma of the adenoid‡, cutaneous melanoma‡ | Fig. 6 and S9 |
| Nose tissue donor 6 | 10270 | Male | 70 | Nasal amputation | Yes | No | Nasal invasive basal cell carcinoma‡ | Fig. 6 and S9 |
| Nose tissue donor 7 | 10928 | Male | 64 | Nasal sinus (median) | Former | No | Low grade chronic nasal inflammation‡, olfactory neuroblastoma | Fig. 6 and S9 |
| Nose tissue donor 8 | 8444 | Male | 45 | Nasal sinus (median) | Passive | No | Nasal squamous cell carcinoma‡ | Fig. 6 and S9 |
| Nose tissue donor 9 | 10378 | Male | 54 | Nasal sinus (left) | No | No | Nasal polymorphic inflammatory infiltration‡, parakeratosis, maxillary | Fig. 6 and S9 |
| Nose tissue donor 10 | 19229 | Female | 58 | Ethmoid right | No | No | Nasal mucormycosis‡, acute myeloid leukemia; diabetus mellitus | Fig. 6 and S9 |

*Details were not provided by the supplier of these cultures. ‡No malignancies were observed in the samples that were used in this study.

**Figures S1 to S9**

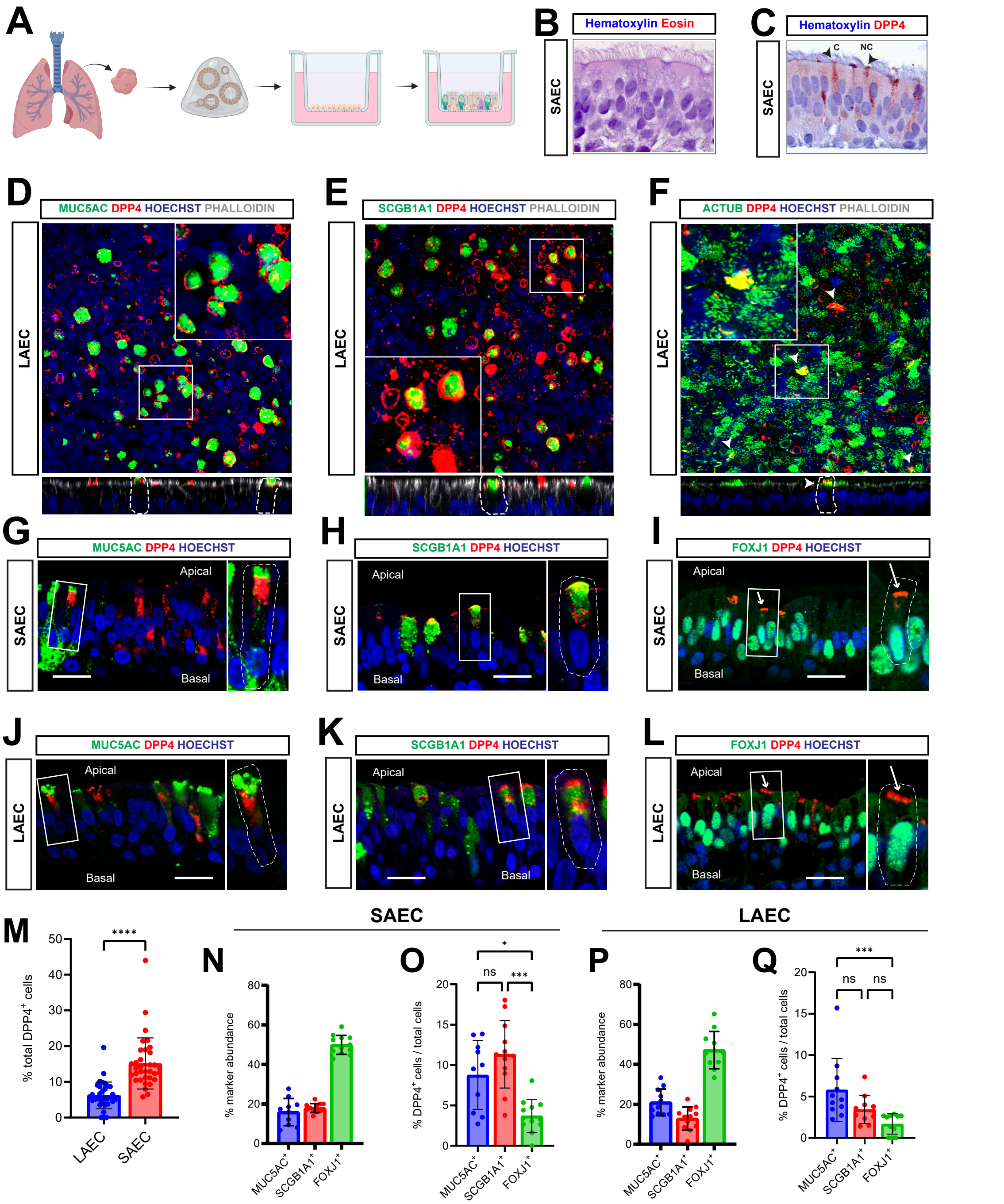


**Figure S1. Characterization of human pulmonary organoid-derived airway cultures.** (A) Schematic overview of human organoid-derived SAECs and LAECs culture and differentiation. Illustration was created with BioRender.com. (B-C) Formalin-fixed and paraffin embedded (FFPE) SAECs were stained for hematoxylin and eosin (B), and IHC was performed for DPP4 (C, red). Images were taken at 20x magnification. Ciliated cells were indicated with "C" and non-ciliated cells with "NC" in the image. (D-F) IF on fixed human LAECs for DPP4 (red) and cell markers (green) MUC5AC (D), SCGB1A1 (E), and ACTUB (F). (G-L) FFPE small and large airway cultures were imaged for DPP4 and cell markers MUC5AC (G, J), SCGB1A1 (H, K) and FOXJ1 (I, L). Inserts (squares) show digital zoom of the original image. (M) DPP4 expression data shown as percentage DPP4+ cells in the total cell population. Data is shown as mean and SD. (N, P) Cell type abundance was quantified in FFPE SAECs (N) and LAECs (P) from 10 images from at least 2 non-sequential tissue sections. Figures show percentage marker abundance from total cells in the image, based on nuclei staining. Data is shown as mean and SD. (O, Q) DPP4 abundance was quantified per cell type from 10 images from at least 2 non-sequential tissue sections. Figures show percentage cells that co-express DPP4 and cell markers from total cells in the image, based on nuclei staining. Data is shown as mean and SD. Significance was tested in M, O, and P, using non-parametric Kruskal-Wallis test with multiple comparisons. *, ***, or **** depicts $p < 0.05$, $p < 0.0005$, or $p < 0.0001$ respectively. Experiments were repeated at least once. Representative images of each staining were shown. Nuclei (blue) were stained with hoechst. F-actin (white) was stained with phalloidin. Scale bars represent 50 μm (D-F) or 20 μm (G-L). Inserts (squares) show digital zoom of the original image.

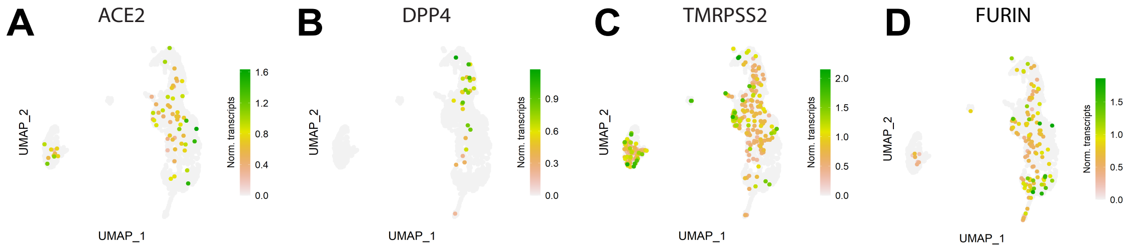


**Figure S2. Expression of coronavirus entry factors in the scRNA-seq dataset.** UMAP plots showing normalized expression of coronavirus entry factors ACE2 (A), DPP4 (B), TMPRSS2 (C) and FURIN (D) in total clustered cells.

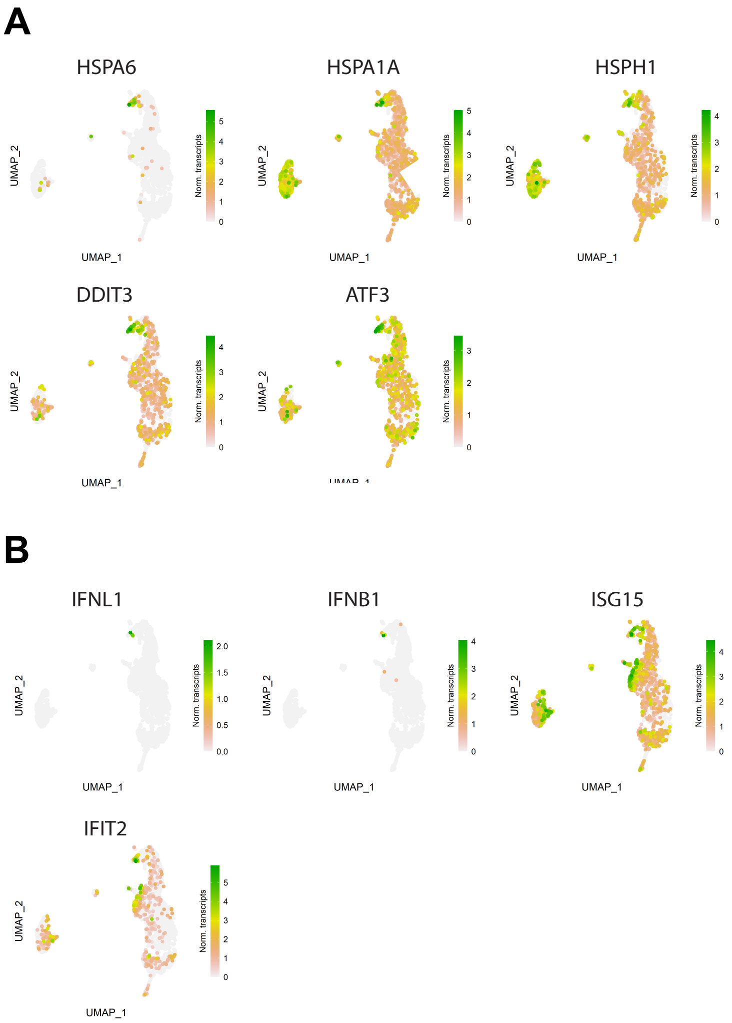


**Figure S3. Expression of ER stress markers and interferon-related markers in the scRNA-seq dataset.** (A) UMAP plots showing normalized expression of ER stress markers in total clustered cells. (B) UMAP plots showing normalized expression of interferon-related markers in total clustered cells.

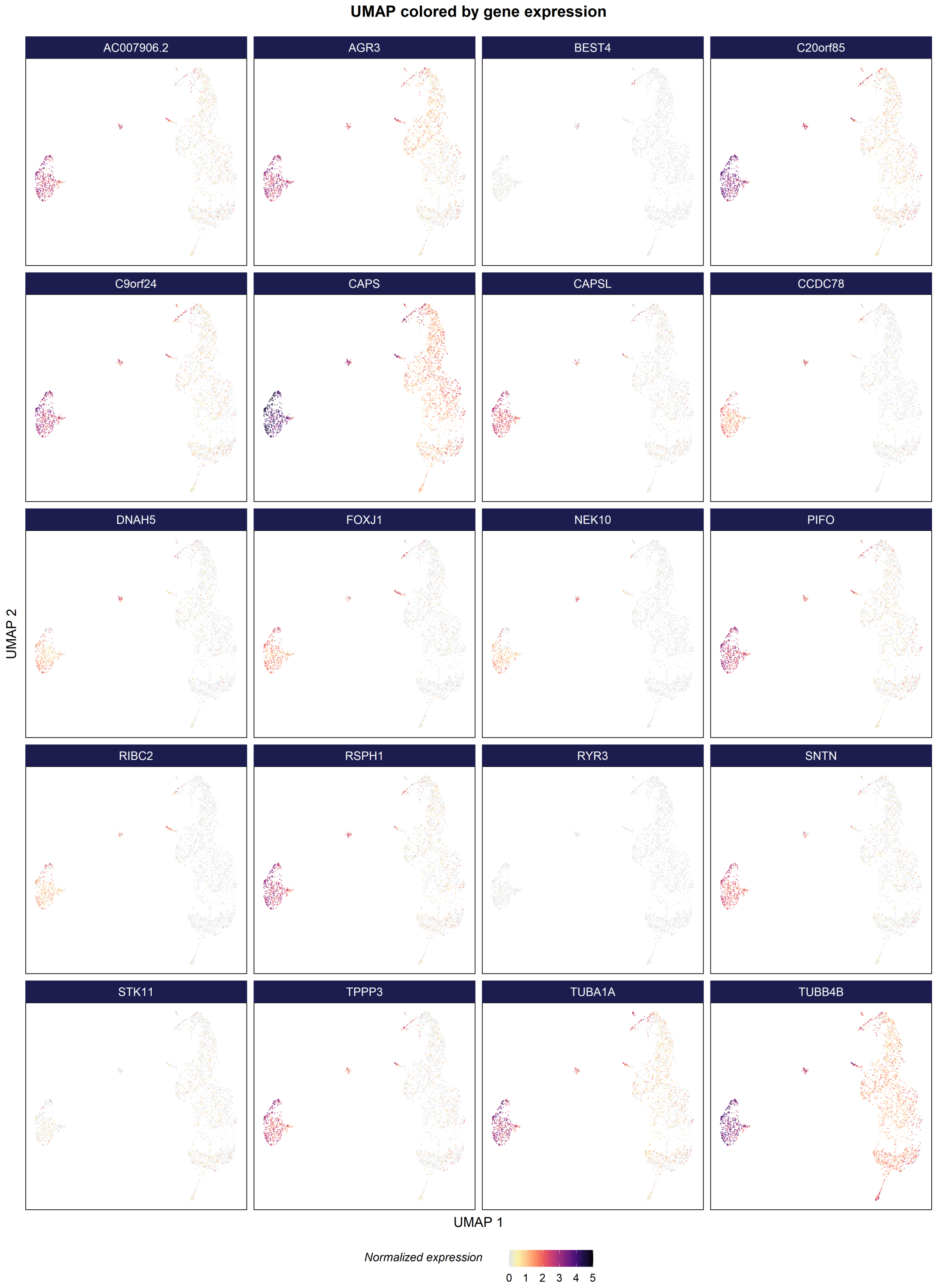


**Figure S4. Expression of multiciliated cell markers in the scRNA-seq dataset.** UMAP plots showing normalized expression of multiciliated cell markers in total clustered cells.

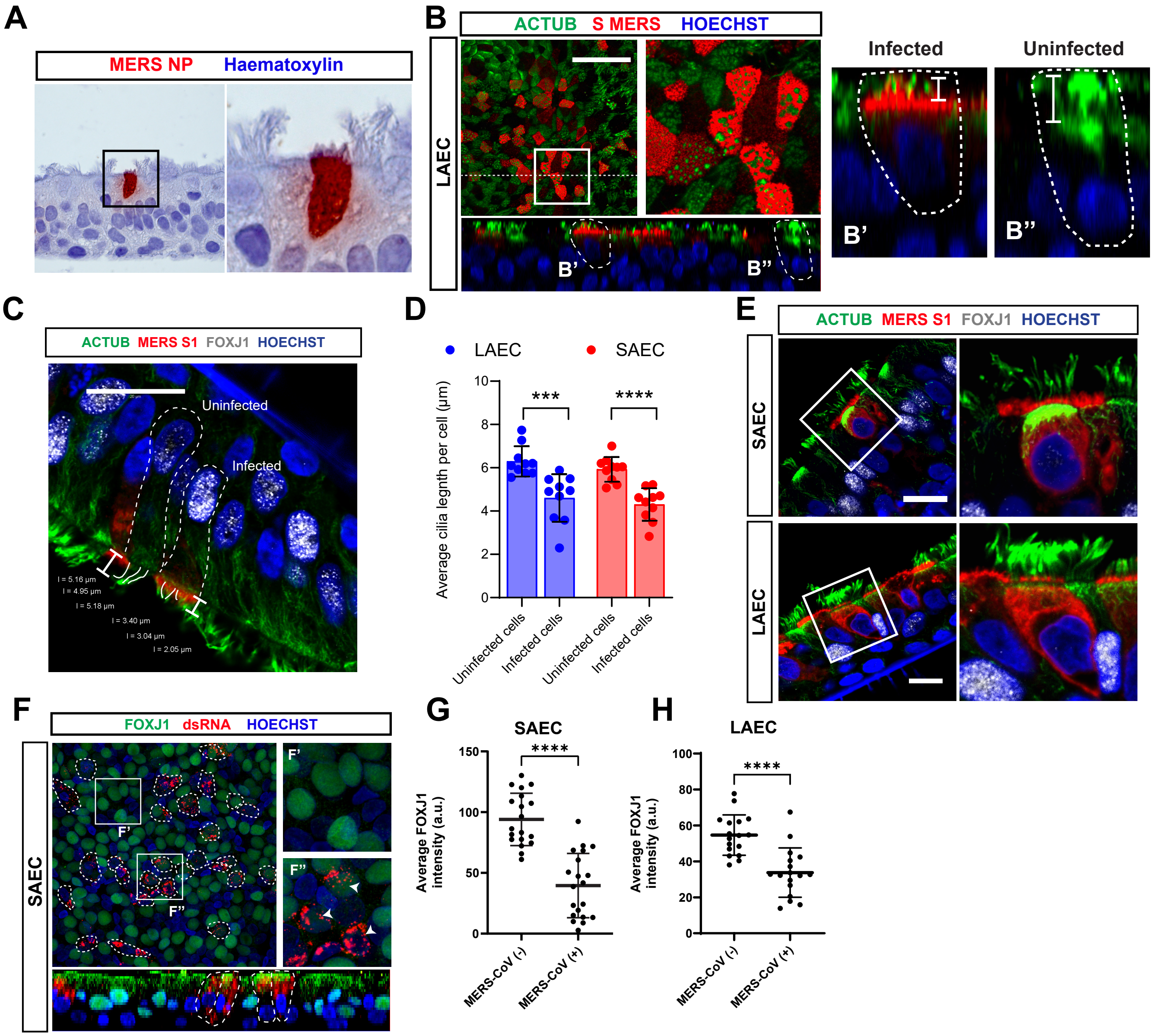


**Figure S5. MERS-CoV infection causes loss of ciliary coverage.** Loss of ciliary coverage was studied at 3 dpi in MERS-CoV infected human SAECs and LAECs. (A) IHC staining of MERS-NP (red) on FFPE infected LAECs at 3 dpi. (B) LAECs were infected with MERS-CoV at moi 0.1 and fixed at 3 dpi for imaging for ACTUB (green) and MERS-S (red). (C-D) Quantification of cilia length in SAECs and LAECs infected with MERS-CoV at 3 dpi. (C) Representative image for cilia length quantification, scale bar depicts 20 μm, length measurements represent true cilia lengths used for quantifications. Quantifications were made by measuring the mean length of three cilia for 10 randomly selected MERS-CoV$^{pos}$ and MERS-CoV$^{neg}$ multiciliated cells. Data depicts mean with SD. Dots represent individual replicates. (E) FFPE SAECs and LAECs were stained for ACTUB (green), MERS-S1 (red) and FOXJ1 (green). (F-H) FOXJ1 expression quantification in MERS-CoV-infected and non-infected cells in small and large airway cultures. (F) Representative image of FOXJ1 quantification. (G-H) Average fluorescent intensity of FOXJ1 in the nuclei of MERS-CoV(-) and MERS-CoV(+) cells were quantified using ZEN software. Data depicts mean and SD of 20 randomly selected cells from one experiment. Significance for D, G and H was tested with unpaired students t-test using Welch's correction. *** or **** depicts $p < 0.005$ or $p < 0.0001$ respectively. Experiments were repeated at least once. Representative images of each staining were shown. Nuclei (blue) were stained with hoechst. Scale bars represent 50 μm. Inserts (squares) show digital zoom of the original image.

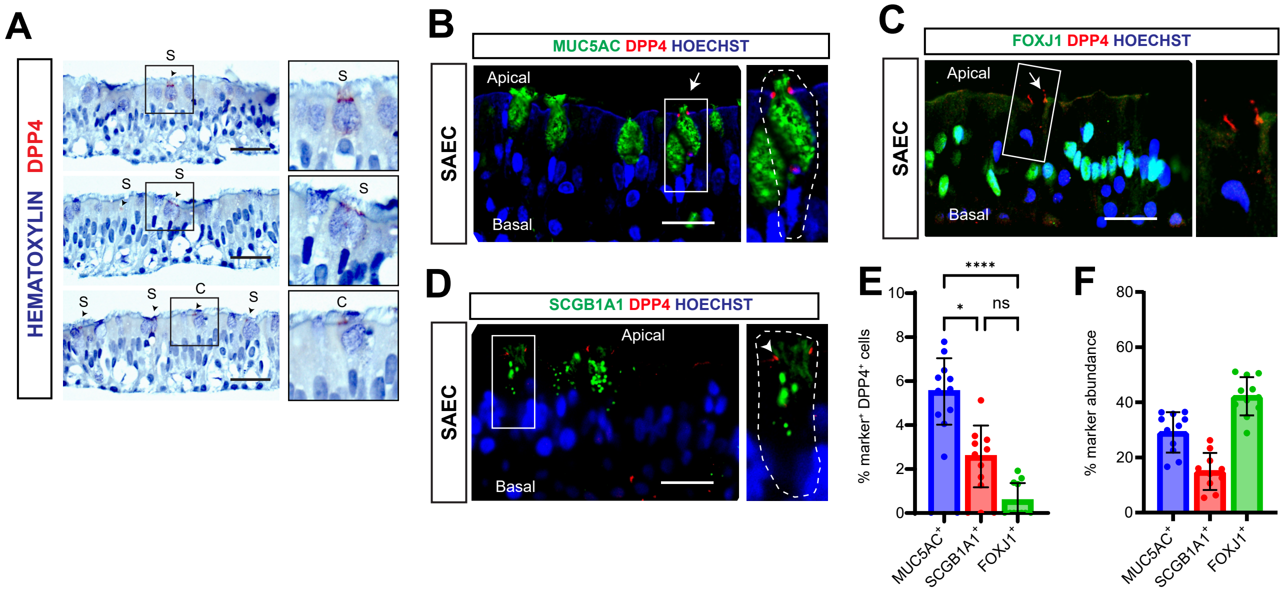


**Figure S6. DPP4 expression characterization in 3-week differentiated pulmonary airway cultures.** (A) 3-week differentiated SAECs were stained for DPP4 with IHC. Three areas of the same insert were shown. Arrows depict DPP4+ cells. Secretory cells were indicated by “S” and multiciliated cells by “C”. (B-D) FFPE 3-week differentiated SAECs were imaged for DPP4 (red) and cell markers (green) MUC5AC (B), SCGB1A1 (C) and FOXJ1 (D) using immunofluorescent staining (IF). Nuclei were stained with hoechst. (E) DPP4 abundance was quantified per cell type from 10 images from at least 2 non-sequential tissue sections. Figures show percentage cells that co-express DPP4 and cell markers from total cells in the image, based on nuclei staining. Data is shown as mean and SD. Significance was tested using non-parametric Kruskal-Wallis test with multiple comparisons. * or **** depicts $p < 0.05$ or $p < 0.0001$ respectively. (F) Percentage cell type abundance was quantified from the same images. Data is shown as mean and SD. Experiments were repeated at least once. Representative images of each staining were shown. Scale bars represent 20 µm. Inserts (squares) show digital zoom of the original image.

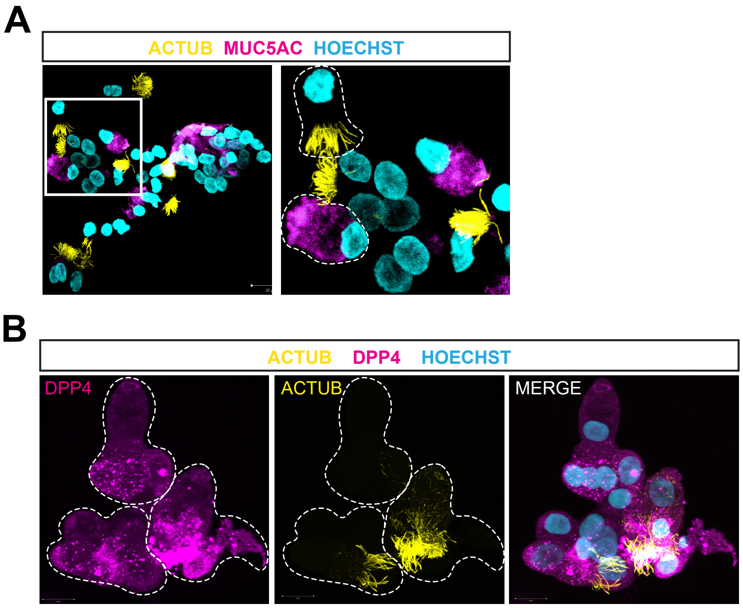


**Figure S7. Characterization of nasal brush material.** (A) Nasal brush material was stained for ACTUB (yellow), MUC5AC (magenta) and Hoechst (cyan). (B) Nasal brush material was stained for ACTUB (yellow), DPP4 (magenta) and Hoechst (cyan). Representative images of each staining were shown. Images were taken at 40x magnification. Inserts (squares) show digital zoom of the original image.

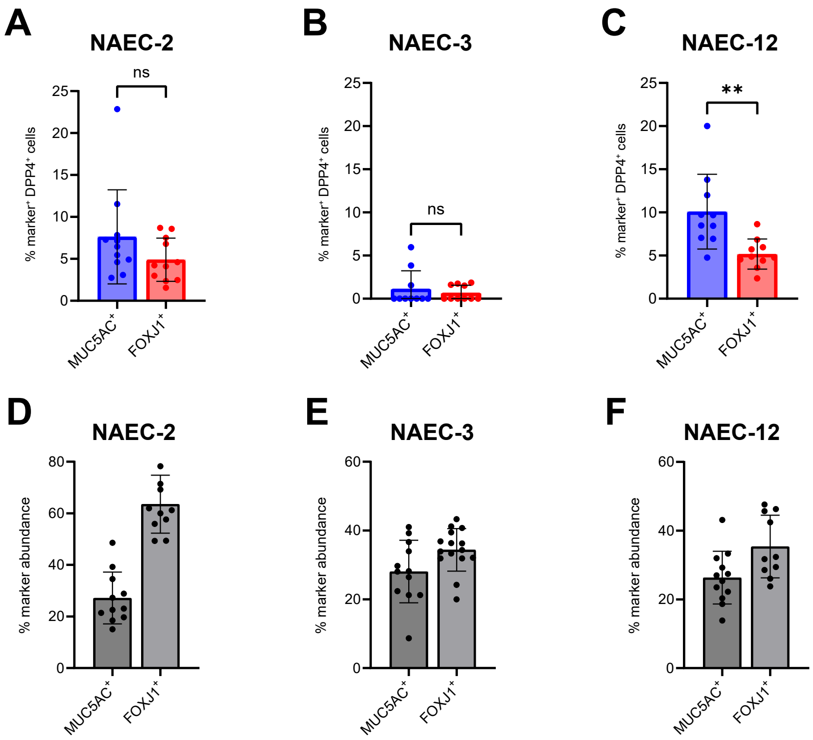


**Figure S8. Characterization of DPP4 expression in nasal cell types.** (A-C) DPP4 abundance was quantified per cell type from 10 images from at least 2 non-sequential tissue sections in NAEC-2, -3, and -12. Figures show percentage cells that co-express DPP4 and cell markers from total cells in the image, based on nuclei staining. Data is shown as mean and SD. Significance was tested using Mann-Whitney U test. ** depicts $p < 0.005$. Some images for NAEC-3 did not contain DPP4. (D-F) Percentage cell marker abundance in the total population was calculated from the same imaging experiments for NAEC-2, -3, and -12.

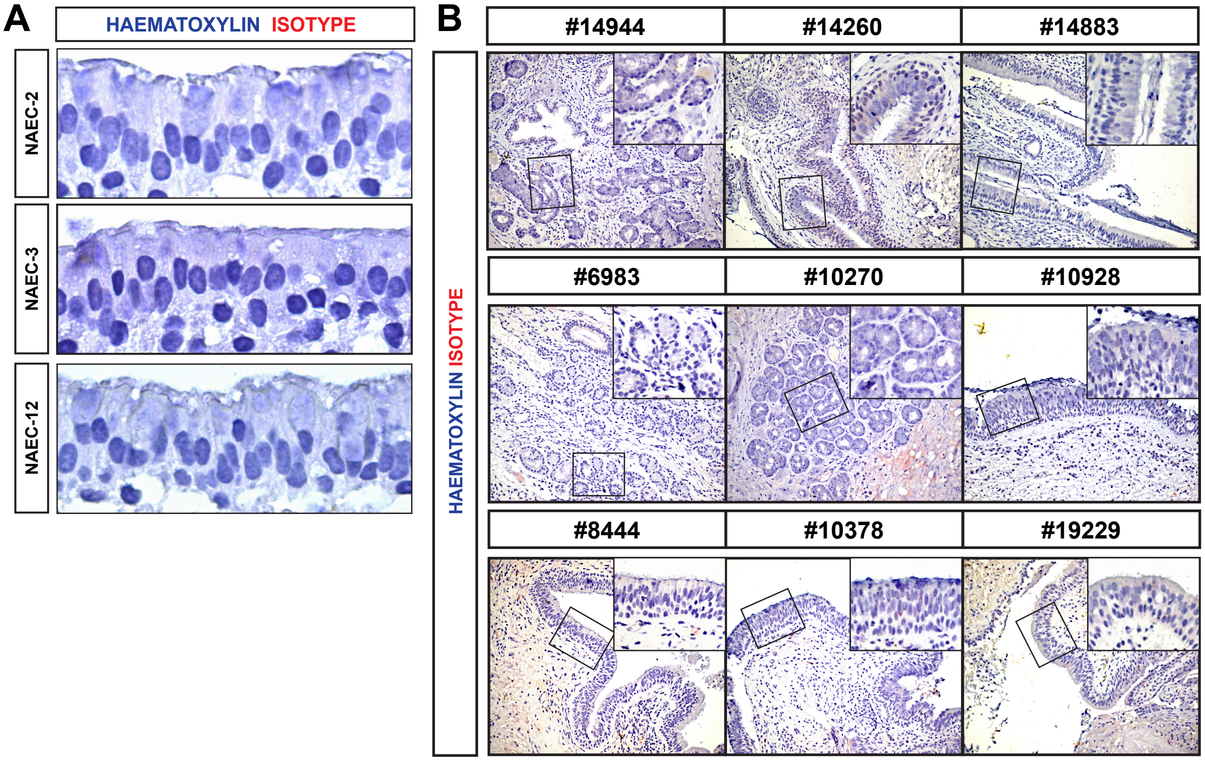


**Figure S9. Isotype control staining of nose donor tissues.** (A-B) Isotype control staining on FFPE NAEC-2, -3, and -12 (A), or FFPE nose tissues from 9 donors (B). Images show respiratory epithelium or submucosal glands at 20x magnification. Representative images of each staining was shown. Inserts (squares) show digital zoom of the original image.

**SI References**


1. N. Sachs *et al.*, Long-term expanding human airway organoids for disease modeling. *EMBO J* **38** (2019).
2. M. M. Lamers *et al.*, An organoid-derived bronchioalveolar model for SARS-CoV-2 infection of human alveolar type II-like cells. *EMBO J* **40**, e105912 (2021).
3. M. C. Chiu *et al.*, Human Nasal Organoids Model SARS-CoV-2 Upper Respiratory Infection and Recapitulate the Differential Infectivity of Emerging Variants. *mBio* **13**, e0194422 (2022).
4. L. W. Rodenburg *et al.*, Protocol for generating airway organoids from 2D air liquid interface-differentiated nasal epithelia for use in a functional CFTR assay. *STAR Protoc* **4**, 102337 (2023).
5. M. M. Lamers *et al.*, SARS-CoV-2 productively infects human gut enterocytes. *Science* **369**, 50-54 (2020).
6. S. van Boheemen *et al.*, Genomic characterization of a newly discovered coronavirus associated with acute respiratory distress syndrome in humans. *mBio* **3** (2012).
7. A. Z. Mykytyn *et al.*, Antigenic cartography of SARS-CoV-2 reveals that Omicron BA.1 and BA.2 are antigenically distinct. *Sci Immunol* **7**, eabq4450 (2022).
8. D. R. Stirling *et al.*, CellProfiler 4: improvements in speed, utility and usability. *BMC Bioinformatics* **22**, 433 (2021).
